# *Mycobacterium tuberculosis* phagosome Ca^2+^ leakage triggers multimembrane ATG8/LC3 lipidation to restrict damage in human macrophages

**DOI:** 10.1126/sciadv.adt3311

**Published:** 2025-03-26

**Authors:** Di Chen, Antony Fearns, Maximiliano G. Gutierrez

**Affiliations:** Host-Pathogen Interactions in Tuberculosis Laboratory, The Francis Crick Institute, 1 Midland Road, London NW1 1AT, UK.

## Abstract

The role of canonical autophagy in controlling *Mycobacterium tuberculosis* (Mtb), referred to as xenophagy, is understood to involve targeting Mtb to autophagosomes, which subsequently fuse with lysosomes for degradation. Here, we found that Ca^2+^ leakage after Mtb phagosome damage in human macrophages is the signal that triggers autophagy-related protein 8/microtubule-associated proteins 1A/1B light chain 3 (ATG8/LC3) lipidation. Unexpectedly, ATG8/LC3 lipidation did not target Mtb to lysosomes, excluding the canonical xenophagy. Upon Mtb phagosome damage, the Ca^2+^ leakage–dependent ATG8/LC3 lipidation occurred on multiple membranes instead of single or double membranes excluding the noncanonical autophagy pathways. Mechanistically, Ca^2+^ leakage from the phagosome triggered the recruitment of the V-ATPase–ATG16L1 complex independently of FIP200, ATG13, and proton gradient disruption. Furthermore, the Ca^2+^ leakage–dependent ATG8/LC3 lipidation limited Mtb phagosome damage and restricted Mtb replication. Together, we uncovered Ca^2+^ leakage as the key signal that triggers ATG8/LC3 lipidation on multiple membranes to mitigate Mtb phagosome damage.

## Teaser

Ca^2+^ leakage triggers LC3 lipidation on multimembrane as Mtb phagosome damage response, independent of autophagy.

## INTRODUCTION

In mammalian cells, membrane Atg8ylation, defined as the lipidation of mammalian autophagy-related protein 8/microtubule-associated proteins 1A/1B light chain 3 (ATG8/LC3) proteins to membranes is a hallmark of autophagy (*1*, *2*). ATG8/LC3 lipidation occurs not only on autophagic structures via canonical autophagy but also on endolysosomes and phagosomes via noncanonical autophagy (*3*–*5*). Xenophagy is a form of selective canonical autophagy that target intracellular bacteria to LC3-positive autophagosomes that will fuse with lysosomes (*6*–*8*). In phagosomes, noncanonical pathways implicating ATG8/LC3 lipidation with no apparent autophagic functions have been referred as LC3-associated phagocytosis (LAP) (*9*).

Damaged endolysosomes can be recognized, degraded, and recycled through a selective canonical autophagy pathway referred as lysophagy, involving ATG8/LC3 lipidation to autophagosomes (*10*–*13*). Alternatively, direct LC3 lipidation to single-membrane damaged endolysosomes can occur in a noncanonical process referred to as conjugation of ATG8 to single membranes (CASM) but the function of the latter is unknown (*14*, *15*). Membrane ATG8/LC3 lipidation can occur on single (*16*) or double membranes, and this difference is used to distinguish between canonical and noncanonical autophagy such as single-membrane ATG8 conjugation (SMAC) or CASM (*2*, *17*).

Organelle proton imbalance has been identified as one of the main triggers of ATG8/LC3 lipidation. Endolysosomal and phagosomal deacidification engages the V1 and V0 subcomplexes of the V-ATPase to control reacidification. This engagement recruits ATG16L1 that, in turn, will control ATG8/LC3 lipidation on the deacidified organelle (*14*, *18*, *19*). The V-ATPase V_1_H subunit binds the ATG16L1 complex in response to proton gradient dissipation (*20*). The dissipation of the proton gradient is primarily associated with channels. However, during organelle damage, other luminal components are leaked into the cytosol in addition to protons. Whether there are other signals during organelle damage that trigger ATG8/LC3 lipidation is poorly understood.

*Mycobacterium tuberculosis* (Mtb) is a human bacterial pathogen known to both induce and evade xenophagy and noncanonical LAP, which both involve the recruitment of LC3 to the Mtb phagosome (*21*–*25*). Mtb damages the phagosome membrane and triggers leakage of phagosome luminal content into the cytosol (*25*). However, the precise mechanisms by which ATG8/LC3 is recruited to Mtb phagosomes and the downstream function of this recruitment in human macrophages remain to be fully defined.

Here, we found that, in human macrophages, Ca^2+^ leakage is a phagosome damage–dependent signal that triggers ATG8/LC3 lipidation. This ATG8/LC3 lipidation is associated with the generation of multiple membranes and no single or double membranes similar to previously described LC3B-positive tubulovesicular structures (LC3-TVS) (*25*). Unexpectedly, ATG8/LC3 lipidation does not target Mtb to lysosomes and Mtb remains in an unacidified LC3-TVS. In contrast, lysosomes fuse with the LC3-TVS, providing a continuous source of membrane. This Ca^2+^ leakage–dependent ATG8/LC3 lipidation contributes to the repair of Mtb damaged membranes in a process that is mechanistically different from canonical autophagy (xenophagy and lysophagy) and noncanonical autophagy (LAP and CASM). Crucially, this phagosome repair mechanism is important for the control of Mtb by macrophages.

## RESULTS

### Ca^2+^ leakage after Mtb phagosome damage triggers ATG8/LC3 lipidation and LC3-TVS formation

**Fig. 1.**
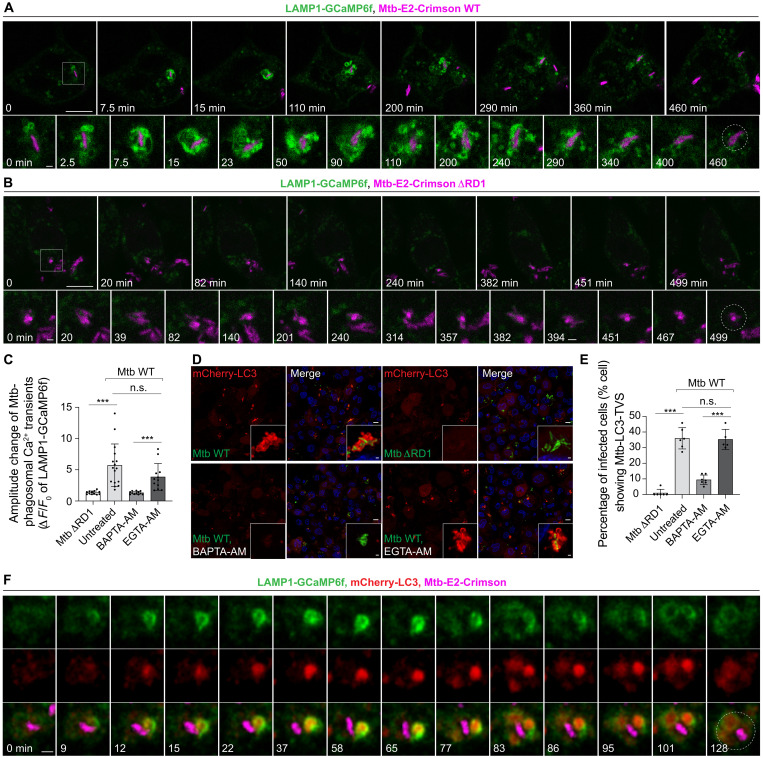
Ca^2+^ leakage after Mtb phagosome damage triggers ATG8/LC3 lipidation. (**A** and **B**) Live-cell imaging sequence showing Ca^2+^ dynamic changes on the Mtb phagosome in THP-1 macrophages stably expressing LAMP1-GCaMP6f during Mtb WT (A) and Mtb ΔRD1 (B) infection. The square indicates the zoomed area. The Mtb phagosome segmentation and live fluorescence signal tracking were analyzed using TrackMate. The dashed circle represents the Mtb area used for LAMP1-GCaMP6f signal quantification. The background areas were random areas in the same cell without Mtb. The live-cell imaging started after 2-hour Mtb uptake. “0” in the series represents the time point for this sequence start. (**C**) Quantification of the amplitude change (Δ*F*/*F*_0_) of Mtb-LAMP1-GCaMP6f signal during 6 hpi under the indicated treatment conditions. Data points correspond to individual Mtb phagosomes = 12 to 16 from 3 to 10 independent experiments. (**D**) THP-1 macrophages stably expressing mCherry-LC3B at 2.5 hpi during Mtb WT and Mtb ΔRD1 infection (MOI: 2) under the indicated treatments. Zoomed-in high-resolution areas are shown in the corner. (**E**) Quantification shows the percentage of infected cells (% cell) showing Mtb-LC3-TVS related to (D). *n* (number of infected cells) = 118 to 163; data points correspond to individual technical replicates from three independent experiments. (**F**) Live-cell imaging sequence showing mCherry-LC3 and LAMP1-GCaMP6f changes during Mtb-WT infection. Images were processed with a Gaussian blur using a sigma (radius) of 1. The dashed circles represent the Mtb area used for mCherry-LC3 and LAMP1-GCaMP6f quantification in fig. S1H. Scale bars, in (A), (B), and (D): 10 μm (main images) and 1 μm (zoomed in); in (F): 1 μm.

Phagosomes contain Ca^2+^ levels that range from 0.4 to 0.6 mM, which is much higher than the resting cytosolic Ca^2+^ concentration of up to 100 nM (*26*–*28*). We hypothesized that, during Mtb phagosome membrane damage, Ca^2+^ leaks out of the phagosome. To visualize rapid Mtb phagosome Ca^2+^ leakage, we generated THP-1 macrophages stably expressing a LAMP1-GCaMP6f probe to detect Ca^2+^ on the cytosolic side of the phagosome as previously described for other organelles (fig. S1, A and B) (*29*, *30*). By live-cell imaging, we observed a rapid increase in LAMP1-GCaMP6f fluorescence surrounding Mtb after phagocytosis (Fig. 1A). In this time series, the continuous Ca^2+^ release triggered by Mtb lasted ~6 hours until bacteria dissociate from the GCaMP6f+ membranes (Fig. 1A, fig. S1C, and movie S1). A quantitative analysis of 100 cells showed that 22% of internalized Mtb wild-type (WT) triggered transient or prolonged Ca^2+^ leakage during the imaging period. The Mtb ESX-1 type 7 secretion system (T7SS) is encoded within the region of difference 1 (RD1) and critical for Mtb phagosome damage (*31*). This Ca^2+^ leakage was membrane damage dependent as the Mtb ΔRD1 mutant did not increase LAMP1-GCaMP6f fluorescence (Fig. 1, B and C; fig. S1C; and movie S2). Mtb phagosomes undergoing Ca^2+^ leakage concomitantly lost LysoTracker Red (LTR) signal, suggesting phagosome leakage of luminal contents (fig. S1, D and E). In some cases, Mtb phagosome Ca^2+^ leakage stopped and the Mtb phagosome recovered LTR, suggesting efficient membrane repair (fig. S1, F and G). 1,2-Bis(2-aminophenoxy)ethane-*N*,*N*,*N*′,*N*′-tetraacetic acid (BAPTA) exhibits faster Ca^2+^ association and dissociation kinetics compared to EGTA (*32*). Therefore, a process that can be affected by BAPTA-acetoxymethyl ester (AM) but not EGTA-AM is likely to involve rapid and localized changes in Ca^2+^ levels rather than steady-state concentrations (*29*). Confirming the Ca^2+^ leakage, treatment with 50 μM BAPTA-AM, but not 50 μM EGTA-AM, abolished the LAMP1-GCaMP6f signal change after Mtb phagosome damage (Fig. 1C). Phagosome damage triggered the formation of Mtb-LC3 positive compartments (Fig. 1, D and E, and fig. S1I), which are tubulovesicular structures surrounding Mtb (Mtb-LC3-TVS) as previously reported (*25*). Live-cell imaging of macrophages coexpressing LAMP1-GCaMP6f and mCherry-LC3B showed that the formation of the Mtb-LC3-TVS occurred after Ca^2+^ leakage (Fig. 1F and fig. S1H). BAPTA-AM but not EGTA-AM treatment markedly impaired the formation of Mtb-LC3-TVS, confirming the role of rapid phagosome Ca^2+^ leakage in triggering LC3 lipidation and LC3-TVS formation (Fig. 1, D and E, and fig. S1I). These observations were confirmed in human induced pluripotent stem cell (iPSC)–derived macrophages (iPSDMs) (fig. S1, J and K). Together, these data show that Mtb phagosome damage triggers ATG8/LC3 membrane conjugation in a Ca^2+^ leakage–dependent manner.

### Ca^2+^ leakage–dependent ATG8/LC3 lipidation does not target Mtb to lysosomes

**Fig. 2.**
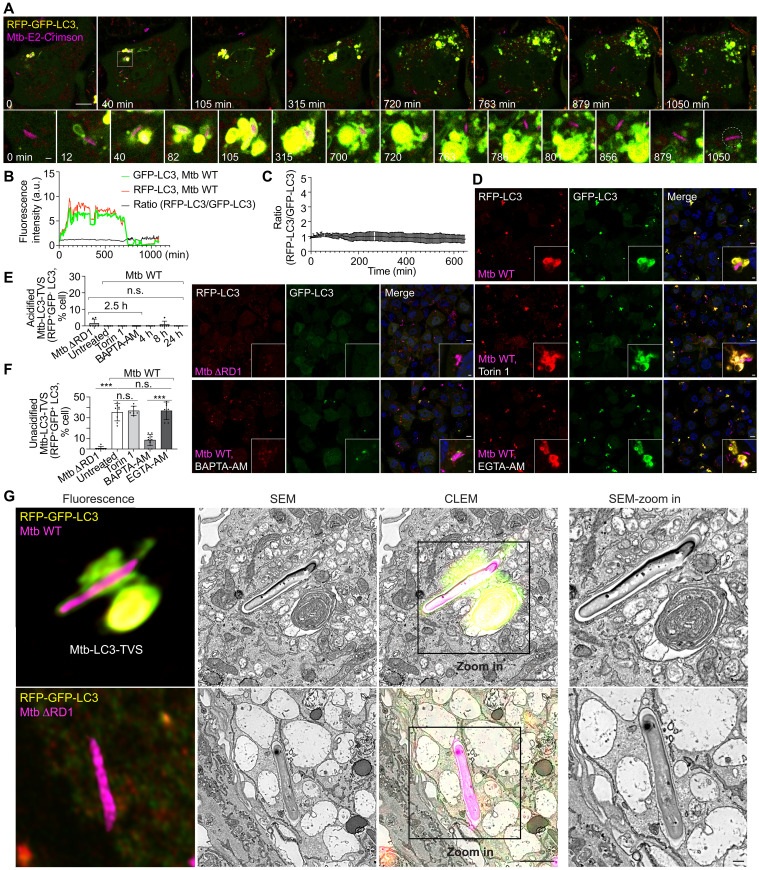
Mtb phagosome damage–dependent Atg8/LC3 multimembrane lipidation does not target Mtb to lysosomes. (**A**) Live-cell imaging sequence showing RFP-GFP-LC3 changes after Mtb infection. White square indicates zoomed-in area. The dashed circle represents the Mtb area used for Mtb-RFP-GFP-LC3 signal quantification in (B). Mtb dissociates from LC3-positive structures from 750 min. (**B**) Ratio change (*F*/*F*_0_) of RFP-LC3 and GFP-LC3 fluorescence intensity and the ratio change of RFP-LC3 (*F*/*F*_0_)/GFP-LC3 (*F*/*F*_0_) in (A). (**C**) Cumulative line scan ratio change in fluorescence intensity of RFP-LC3 (*F*/*F*_0_) to GFP-LC3 (*F*/*F*_0_) surrounding Mtb. *n* = 3. (**D**) Mtb WT and Mtb ΔRD1 infection (MOI: 2) in THP-1 macrophages stably expressing RFP-GFP-LC3B at 2.5 hpi under indicated treatments. (**E**) Quantification shows the percentage of infected cells (MOI: 2) showing RFP-LC3–positive and GFP-LC3–negative Mtb-LC3-TVS under indicated treatments and different postinfection time points related to (D). *n* (number of infected cells) = 97 to 195; data points correspond to individual technical replicates from three independent experiments. h, hours. (**F**) Quantification shows the percentage of infected cells showing both RFP-LC3– and GFP-LC3–positive Mtb-LC3-TVS related to (D). *n* (number of infected cells) = 105 to 195; data points correspond to individual technical replicates from three independent experiments. (**G**) CLEM analysis shows the RFP-LC3– and GFP-LC3–positive Mtb-LC3-TVS (2.5 hpi, MOI: 2) and LC3-negative phagosome during Mtb ΔRD1 infection. SEM, scanning electron microscopy. Scale bars, in (A) and (D): 10 μm (main images) and 1 μm (zoomed-in area); in (G): 1 μm (main images) and 200 nm (zoomed-in area).

We then analyzed whether the Ca^2+^ leakage–dependent LC3 membrane lipidation was a xenophagy response that targeted bacteria to lysosomes. Macrophages expressing the reporter red fluorescent protein (RFP)–green fluorescent protein (GFP)–LC3B were infected with Mtb to monitor autophagic flux (*33*). In macrophages, there was a basal level population of RFP-positive and GFP-negative LC3 (RFP^+^GFP^−^LC3) compartments (fig. S2A) and the mTOR inhibitor Torin-1 induced an increase in RFP^+^GFP^−^LC3 autolysosomes (fig. S2, A and B). In contrast, the V-ATPase inhibitor bafilomycin A1 (BafA1) led to the accumulation of RFP^+^GFP^+^LC3 autophagosomes (fig. S2, A and B). Infection with Mtb WT but not with Mtb ΔRD1 resulted in the formation of RFP^+^GFP^+^LC3-TVS in both THP-1 macrophages and iPSDMs (Fig. 2, A to E; fig. S2, C to F; and movie S3). BAPTA-AM but not EGTA-AM treatment blocked the formation of Mtb-RFP^+^GFP^+^LC3-TVS, indicating that this process was Ca^2+^ leakage dependent (Fig. 2, D and F). Unexpectedly, these newly formed Mtb compartments remained RFP^+^GFP^+^LC3 even after 24 hours of infection or Torin-1 treatment in both THP-1 macrophages and iPSDMs, indicating that these compartments do not acidify (Fig. 2, D to F; fig. S2, C to F; and movie S3). To better understand the nature of these nonacidic Mtb-LC3-TVS, we used correlative light electron microscopy (CLEM). Mtb-LC3-TVS were multimembrane structures that contained small vesicles and were different from the typical double-membrane autophagosomes or single-membrane LAPosomes (Fig. 2G). As expected, Mtb WT, but not the Mtb ΔRD1 strain, induced multimembrane formation (Fig. 2G). Together, these data indicate that LC3-TVS are unlikely to be part of the canonical pathway of autophagosomes undergoing acidification and degradation (e.g., lysophagy or xenophagy). Moreover, LC3-TVS were not composed of single membranes excluding noncanonical pathways (e.g., LAP or CASM). Thus, Mtb phagosome Ca^2+^ leakage triggers the formation of unacidified multimembrane LC3-TVS.

### Fusion of endolysosomes provides the Mtb-LC3-TVS membrane source

**Fig. 3.**
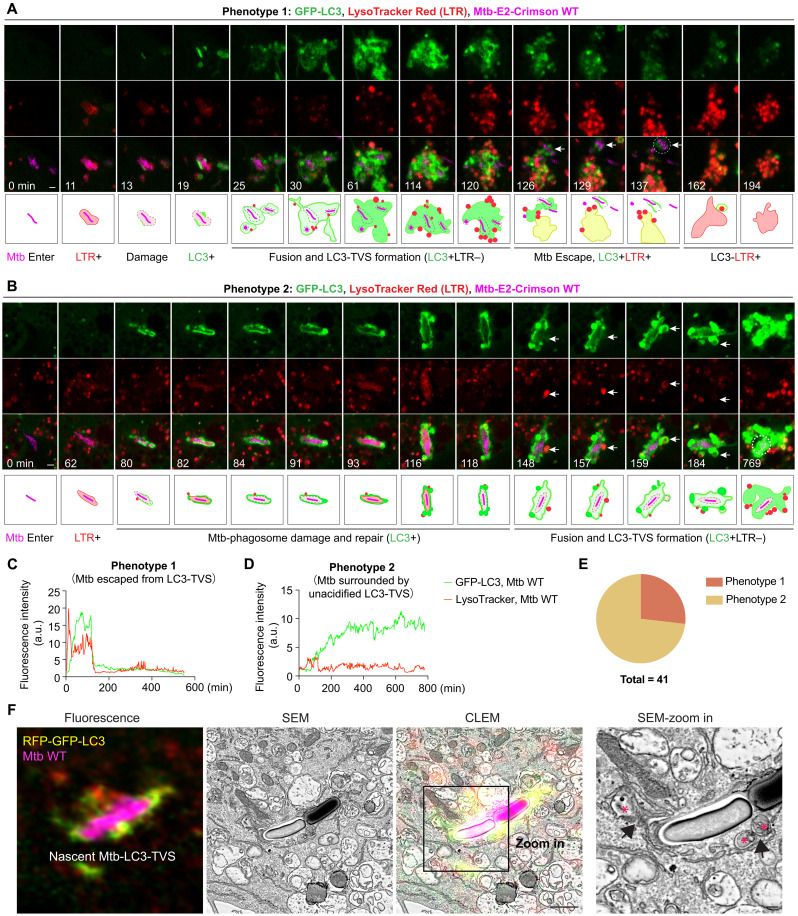
Fusion of endolysosomes with Mtb LC3+ phagosomes provides multiple membrane sources. (**A**) Live-cell imaging sequence and diagram showing GFP-LC3 and LTR changes during Mtb-WT infection. After Mtb phagosome damage, Mtb segregated (from 126 min) and the remaining LC3-positive compartment underwent acidification. Dashed circle represents the Mtb area used for LTR signal quantification in (C). (**B**) Live-cell imaging sequence and diagram showing GFP-LC3 and LTR changes during Mtb-WT infection. After Mtb phagosome damage, Mtb remained in GFP-LC3–positive compartments (Mtb-LC3-TVS). Mtb-LC3-TVS continuously fused with endolysosomes without acidification. Dashed circle represents the Mtb area used for Mtb compartment LTR signal quantification in (D). (**C**) Ratio change (*F*/*F*_0_) of GFP-LC3 and LTR fluorescence intensity surrounding Mtb in (A). (**D**) Ratio change (*F*/*F*_0_) of GFP-LC3 and LTR fluorescence intensity surrounding Mtb in (B). (**E**) Percentage of Phenotype 1 and Phenotype 2 related to (A) and (B), n = 41 Mtb-LC3 positive compartments. This analysis includes all observed instances of Mtb-triggered LC3-TVS formation captured over 48 hours of live-cell imaging. (**F**) CLEM analysis shows a nascent Mtb-LC3-TVS interacting (arrowhead) with small vesicles (indicated by *) in THP-1 macrophages stably expressing RFP-GFP-LC3B during Mtb WT infection (2.5 hpi, MOI: 2). Scale bars, in (A) and (B): 1 μm; in (F): 1 μm (main images) and 200 nm (zoomed-in area).

To study the formation and fate of these unacidified Mtb-LC3-TVS, we traced single GFP-LC3B expressing macrophages labeled with LTR for 48 hours by high-resolution live-cell imaging. Two distinct phenotypes were observed. In the first phenotype, Mtb phagosome acquired LTR at 11 min (Fig. 3A and movie S4) followed by ATG8/LC3 conjugation at 25 min with LTR leakage, indicative of phagosome damage (Fig. 3A and movie S4). Although there was a continuous fusion of Mtb phagosomes with LTR+ endolysosomes, these LC3+ compartments did not mature into a GFP-LC3–negative (LC3−) or LTR+ compartments (Fig. 3A and movie S4). At later stages, Mtb segregated from these LC3-TVS that now fused with LTR+ endolysosomes with a reduction in the GFP-LC3 signal and increase in LTR signal in 60 min (Fig. 3, A to C, and movie S4). In the second phenotype, the Mtb phagosome acquired LTR at 62 min, followed by LC3 conjugation and LTR leakage at 80 min (Fig. 3, B to D, and movie S5). There was a transient recovery as well as LTR loss and recovery from 82 to 116 min, suggesting membrane repair associated with continuous membrane damage (Fig. 3, B and D, and movie S5). The Mtb phagosome leaked LTR without further recovery from 118 min onward. The LC3+ Mtb compartments continuously fused with LTR+ endolysosomes although remained GFP-LC3+ and LTR− for around 30 hours (Fig. 3, B and D, and movie S5). From 150 to 161 min, LTR+ endolysosomes fused with the damaged Mtb compartment followed by LC3 lipidation and loss of LTR signal, suggesting that these LTR+ endolysosomes contributed to the generation of ATG8/LC3+ multiple membranes (Fig. 3, B and D, and movie S5). From 380 to 430 min, there was a GFP-LC3+ vesicle segregated from Mtb-LC3-TVS that became LTR+ and gradually lost GFP-LC3 at 40 min (fig. S2G and movie S5). Meanwhile, damaged Mtb-LAMP1-GCaMP6f+ compartments are continuously surrounded and fuse with GCaMP6f+ vesicles, suggesting that endolysosomes are a source of Ca^2+^ (Fig. 1A and movie S1). We traced 41 Mtb phagosomes by live-cell imaging during 48 hours, 11 events of phenotype 1 whereas 30 events of phenotype 2 were observed (Fig. 3E). Our analysis showed that ~61% of Mtb-LC3-TVS structures (of 108 analyzed) colocalized with CD63 (fig. S2H). Confirming these observations, a CLEM analysis showed that the nascent, unacidified Mtb-LC3-TVS (RFP^+^GFP^+^LC3) were surrounded by multiple endocytic vesicles fusing with the Mtb LC3+ compartment (Fig. 3F, arrows). Together, these data show that the lack of acidification was not caused by blockage of fusion between LC3+ Mtb compartments and endolysosomes. Endolysosomes continuously interact with the damaged Mtb phagosomes, providing a new membrane that is subsequently conjugated to ATG8/LC3.

### Formation of Mtb-LC3-TVS is independent of canonical autophagy

To define whether canonical autophagy was involved in the Ca^2+^ leakage–dependent Mtb-LC3-TVS formation, we generated a series of autophagy gene knockout (KO) THP-1 macrophages (see Materials and Methods). The ULK complex containing FIP200-ATG13 serves as a master regulator of canonical autophagy initiation by triggering the recruitment of the PI3K (phosphatidylinositol 3-kinase) III complex and the ATG8/LC3 lipidation system under nutrient deprivation conditions (*34*, *35*). ATG16L1 and ATG7 are core components of the ATG8/LC3 lipidation system involved in canonical and noncanonical autophagy pathways (*36*). As expected, the autophagic substrate p62 accumulated in *FIP200*, *ATG13*, *ATG7*, and *ATG16L1* KO macrophages compared to WT macrophages (fig. S2I) and the formation of RFP^+^GFP^−^LC3B puncta was blocked in *FIP200*, *ATG13*, *ATG7*, and *ATG16L1* KO macrophages after Torin-1 treatment (fig. S2, J and K). Furthermore, the turnover of LC3-I to LC3-II was blocked in *ATG16L1 KO* and *ATG7* KO macrophages but not in *FIP200* KO and *ATG13* KO macrophages (fig. S2I).

**Fig. 4.**
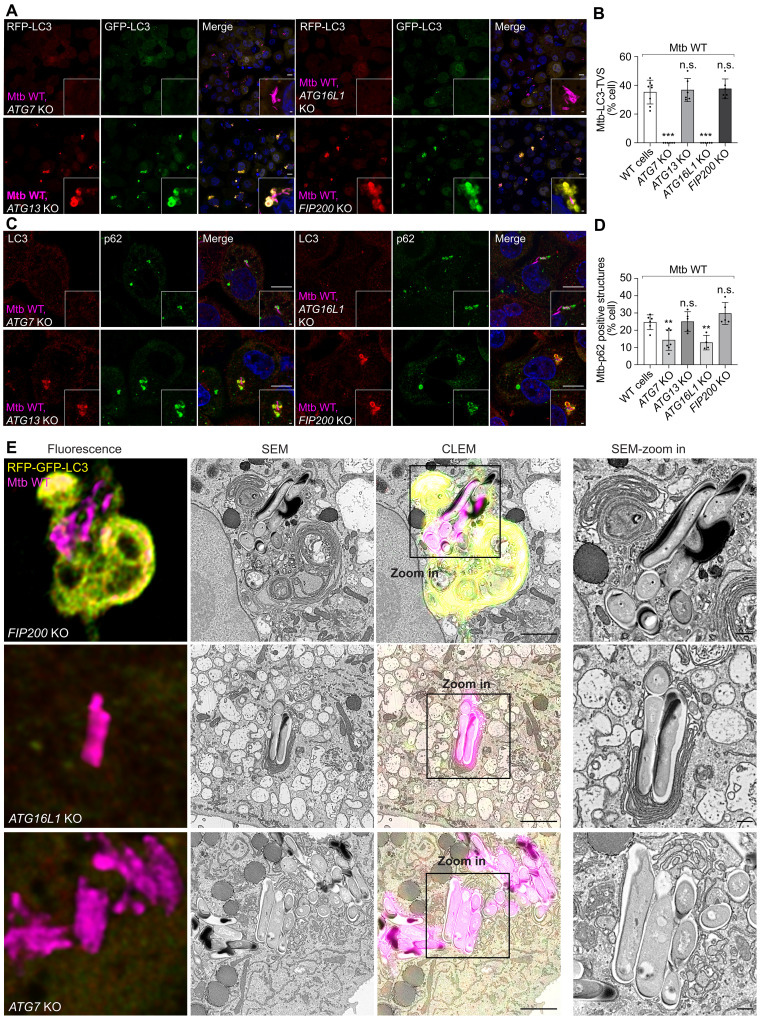
Ca^2+^ leakage–dependent ATG8/LC3 lipidation requires the ATG16L1-LC3 lipidation system but not the FIP200-ATG13 complex. (**A**) Mtb WT infection (MOI: 2) in *ATG7* KO, *ATG16L1* KO, *ATG13* KO, and *FIP200* KO THP-1 macrophages stably expressing RFP-GFP-LC3B at 2.5 hpi. (**B**) Quantification shows the percentage of infected cells showing Mtb-LC3-TVS in WT and *ATG7* KO, *ATG16L1* KO, *ATG13* KO, and *FIP200* KO cells related to (A). The dataset for WT condition matches data in Fig. 2F. *n* (number of infected cells) = 80 to 195; data points correspond to individual technical replicates from three independent experiments. (**C**) p62 and LC3 staining in *ATG7* KO, *ATG16L1* KO, *ATG13* KO, and *FIP200* KO THP-1 macrophages infected with Mtb WT at 2.5 hpi. (**D**) Percentage of infected cells showing Mtb-p62–positive structures in WT and *ATG7* KO, *ATG16L1* KO, *ATG13* KO, and *FIP200* KO cells related to (C) and fig. S2L. *n* (number of infected cells) = 80 to 185; data points correspond to individual technical replicates from three independent experiments. (**E**) CLEM shows the Mtb-LC3-TVS in *FIP200* KO THP-1 macrophages and LC3-negative membrane surrounding Mtb in *ATG7* and *ATG16L1* KO THP-1 macrophages during Mtb WT infection (2.5 hpi, MOI: 2). Scale bars, in (A) and (C): 10 μm (main images) and 1 μm (inserted area); in (E): 1 μm (main images) and 200 nm (enlarged area).

After Mtb infection, LC3-TVS formation was abolished in *ATG16L1* KO and *ATG7* KO macrophages but not in *ATG13* and *FIP200* KO macrophages (Fig. 4, A and B). The autophagy adaptor p62 was recruited to LC3-TVS in WT macrophages (fig. S2L) and ATG8/LC3 conjugation, but p62 recruitment was severely impaired in both *ATG7* KO and *ATG16L1* KO macrophages. In contrast, in *FIP200* or *ATG13* KO macrophages, LC3-TVS formation was similar when compared to WT macrophages (Fig. 4, B to D, and fig. S2L). Notably, Mtb-p62–positive structures were smaller and compacted in *ATG7* KO and *ATG16L1* KO macrophages compared to WT, *FIP200* KO, or *ATG13* KO macrophages (Fig. 4, C and D, and fig. S2L). Thus, the multimembrane LC3-TVS generation depends on the ATG8/LC3 conjugation machinery but not p62 recruitment. A three-dimensional (3D) CLEM analysis showed that *FIP200* depletion had no effect on Mtb-LC3-TVS formation (Fig. 4E and movie S6), whereas *ATG16L1* and *ATG7* KO changed the ultrastructural organization of membranes associated with Mtb (Fig. 4E). These LC3− membranes were compacted when compared to LC3+ membranes in WT and *FIP200* KO macrophages (Figs. 2F and 4E). We concluded that multimembrane Mtb-LC3-TVS formation requires the ATG8/LC3 lipidation machinery independently of canonical autophagy in response to membrane damage but not part of canonical selective autophagy.

### Ca^2+^ leakage triggers GAL8 recruitment to the Mtb-LC3-TVS

**Fig. 5.**
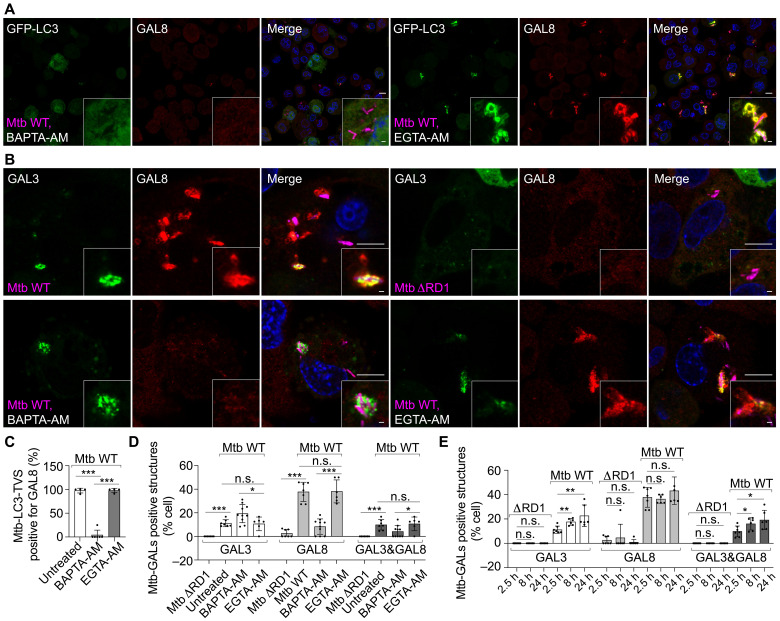
GAL8 is recruited to the Mtb-LC3-TVS during early and late damage. (**A**) GAL8 staining in THP-1 macrophages stably expressing GFP-LC3B after Mtb WT infection (2.5 hpi, MOI: 2) under treatment with BAPTA-AM and EGTA-AM. (**B**) GAL8 and GAL3 staining in THP-1 macrophages after Mtb WT and Mtb ΔRD1 infection (2.5 hpi, MOI: 2) under indicated treatments. (**C**) Percentage of Mtb-LC3-TVS positive for GAL8 related to (A) and fig. S3D. *n* (number of infected cells) = 51 to 72; data points correspond to individual technical replicates from three independent experiments. (**D**) Percentage of infected cells with Mtb-GAL3 positive, Mtb-GAL8 positive, and Mtb-GAL3/GAL8 double positive compartments. *n* (number of infected cells) = 96 to 151; data points correspond to individual technical replicates from three independent experiments. (**E**) Percentage of infected cells showing the Mtb-GAL3 positive, Mtb-GAL8 positive, and Mtb-GAL3/GAL8 double positive structures at 2.5, 8, and 24 hpi. *n* (number of infected cells) = 101 to 151; data points correspond to individual technical replicates from three independent experiments. The datasets for 2.5 hpi matches data from (D). Scale bars, in (A) and (B): 10 μm (main images) and 1 μm (inserted area).

Galectin-3 (GAL3) is a well-established marker of damaged phagosomes (*37*). We found that most of the Mtb-GAL3–positive structures were LC3+ (fig. S3, A to C). However, a fraction of LC3-TVS were negative for GAL3 (fig. S3, A to C). Given that GAL3 does not label all the damaged phagosomes (*38*), we tested Galectin-8 (GAL8), another galectin reported to be recruited after membrane damage (*39*). Notably, GAL8 localized to the Mtb-LC3-TVS (Fig. 5C and fig. S3D), whereas GAL3 formed puncta-like structures and was mostly recruited into the lumen of the LC3-TVS (fig. S3A). Most of the GAL3+ structures localized inside GAL8+ membranes (Fig. 5, A and B, and fig. S3E). A quantitative analysis showed that most of the LC3-TVS were positive for GAL8 (Fig. 5C), and there were more Mtb phagosomes positive for GAL8 than GAL3 during 24 hours of infection. In addition, a higher proportion of Mtb phagosomes were double positive for GAL3 and GAL8 after 8 hours postinfection (hpi) (Fig. 5, D and E). Notably, BAPTA-AM, but not EGTA-AM, abolished the recruitment of GAL8 but not GAL3 (Fig. 5, B and D). These observations were confirmed in human iPSDMs (fig. S3, F and G). Furthermore, live-cell imaging showed that Mtb-phagosomal Ca^2+^ leakage is closely associated with GAL8 recruitment (fig. S3, H and I). Together, these results show that Ca^2+^ leakage–dependent ATG8/LC3 conjugation during limited Mtb phagosome damage is initially recognized by GAL8 followed by extensive membrane damage that is recognized by both GAL8 and GAL3.

### Ca^2+^ leakage–dependent ATG8/LC3 lipidation requires V-ATPase–ATG16L1 complex independently of proton gradient disruption

**Fig. 6.**
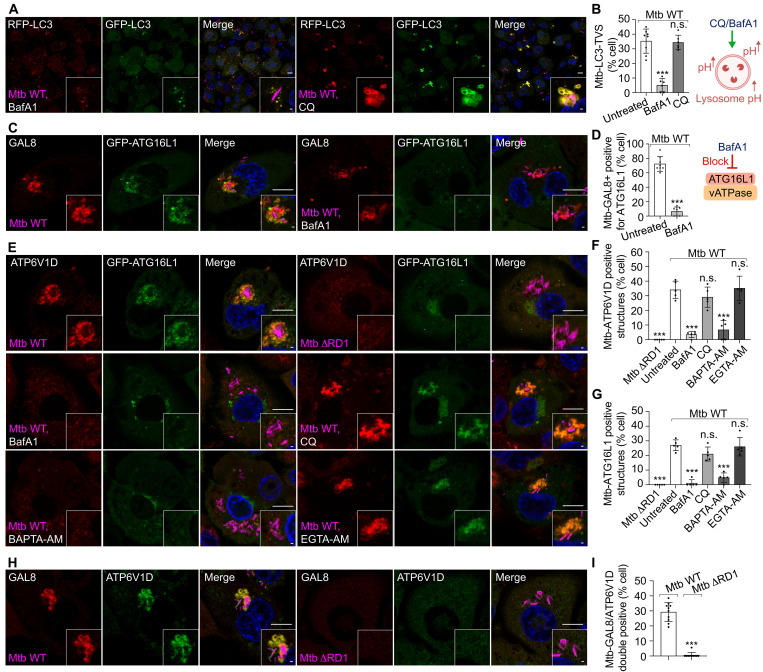
Ca^2+^ leakage triggers the recruitment of V-ATPase–ATG16L1 to Mtb-LC3-TVS. (**A**) Mtb infection (MOI: 2) in THP-1 macrophages stably expressing RFP-GFP-LC3B at 2.5 hpi under BafA1 and CQ treatment. (**B**) Percentage of infected cells showing Mtb-LC3-TVS under BafA1 and CQ treatment, related to (A). The dataset for control cells matches data from Fig. 2F. Diagram shows endolysosome pH after BafA1and CQ treatment. *n* (number of infected cells) = 122 to 195; data points correspond to individual technical replicates from three independent experiments. (**C**) GAL8 staining in THP-1 macrophages stably expressing GFP-ATG16L1 at 2.5 hpi during Mtb infection (MOI: 2) under BafA1 treatment. (**D**) Quantification shows the percentage of Mtb-GAL8 positive structures positive for ATG16L1 under BafA1 treatment, related to (C). Diagram shows the function of BafA1 in blocking VATPase-ATG16L1 complex assembly. *n* (number of infected cells) = 178 and 202; data points correspond to individual technical replicates from 3 independent experiments. (**E**) ATP6V1D staining in THP-1 macrophages stably expressing GFP-ATG16L1 at 2.5 hpi during Mtb WT and Mtb ΔRD1 infection (MOI: 2) under indicated treatments. (**F** and **G**) Percentage of infected cells showing Mtb-ATP6V1D positive (F) and Mtb-ATG16L1 positive (G) structures under the indicated treatments, related to (E). *n* (number of infected cells) = 93 to 130; data points correspond to individual technical replicates from three independent experiments. (**H**) GAL8 and ATP6V1D staining in THP-1 macrophages infected with Mtb WT and Mtb ΔRD1 at 2.5 hpi. (**I**) Percentage of Mtb WT– and Mtb ΔRD1–infected cells showing the GAL8– and ATP6V1D–double positive structures, related to (H). *n* (number of infected cells) = 215 and 175; data points correspond to individual technical replicates from three independent experiments. Scale bars, 10 μm (main images) and 1 μm (inserted area).

The activation of the lysosomal Ca^2+^ channel induces LC3 lipidation on lysosomal membranes that requires V-ATPase–ATG16L1 assembly (*40*). We tested whether V-ATPase–ATG16L1 assembly was implicated in the Ca^2+^ leakage–dependent ATG8/LC3 conjugation. The lysosomotropic agent chloroquine (CQ) neutralizes lysosomes by accumulating inside and sequestering protons, without directly inhibiting the assembly of V-ATPase (*41*). We found that CQ neutralized lysosomes much faster than BafA1 in human macrophages (fig. S4, A to C). However, treatment with V-ATPase inhibitor BafA1, but not with CQ, blocked Ca^2+^ leakage–dependent Mtb-LC3-TVS formation (Fig. 6, A and B, and fig. S4, D to F). During V-ATPase assembly, the cytosolic V1 subunits are recruited to the phagosome membrane V0 subunits (*42*). The V-ATPase V1 subunits ATP6V1D and ATG16L1 were both recruited together with GAL8 to the Mtb-LC3-TVS (Fig. 6, C to I, and fig. S4, G and H). BAPTA-AM and BafA1, but not EGTA-AM and CQ, abolished the assembly of the ATP6V1D-ATG16L1 complex and LC3 lipidation (Fig. 6, A to I, and fig. S4, D to H). Depletion of *ATG16L1* and *ATG7*, which impairs Mtb-LC3-TVS formation, had no effect on the assembly of ATP6V1D to Mtb-LC3-TVS (fig. S4, I and J). Together, these data show that Mtb phagosome Ca^2+^ leakage triggers an acidification-dependent assembly of V-ATPase, which then recruits ATG16L1.

### Ca^2+^ leakage–dependent LC3-TVS restrict Mtb phagosome damage and Mtb replication

**Fig. 7.**
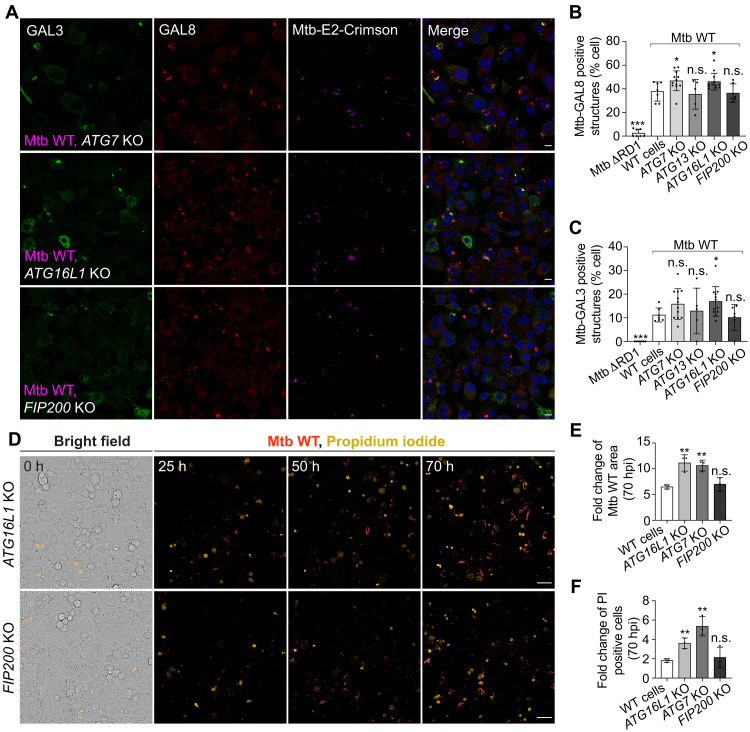
ATG8/LC3 lipidation restricts phagosome damage and Mtb infection. (**A**) GAL8 and GAL3 staining in WT and *ATG16L1* KO and *FIP200* KO THP-1 macrophages after Mtb WT and Mtb ΔRD1 infection (2.5 hpi, MOI: 2). (**B** and **C**) Percentage of infected cells showing the Mtb-GAL8 positive and Mtb-GAL3 positive structures in WT and *ATG7* KO, *ATG16L1* KO, *ATG13* KO, and *FIP200* KO THP-1 macrophages related to (A) and fig. S5A. The datasets for WT cells are same as Fig. 5D. *n* (number of infected cells) = 96 and 174; data points correspond to individual technical replicates from three independent experiments. (**D**) Representative micrographs at indicated time points of *ATG16L1* KO and *FIP200* KO THP-1 macrophages infected with Mtb WT (red) in the presence of PI+ necrotic cells (yellow). Bright-field images show the localization of macrophages. Data are representative from one of three independent experiments. (**E**) Quantification shows the fold change of Mtb area at 70 hpi with WT Mtb in WT, *ATG7* KO, *ATG16L1* KO, and *FIP200* KO THP-1 macrophages. Data are from three independent biological replicates, each of which represents the mean of three technical replicates. (**F**) Quantification shows the fold change in the number of PI-positive cells 70 hpi with WT Mtb, compared to noninfected cells, in WT, *ATG7* KO, *ATG16L1* KO, and *FIP200* KO THP-1 macrophages. Data are from three independent biological replicates, each of which represents the mean of three technical replicates. Scale bars, in (A): 10 μm; in (D): 50 μm.

An increase in GAL3+ Mtb was observed after BAPTA-AM and BafA1 treatment, suggesting that Ca^2+^ leakage–dependent ATG8/LC3 lipidation is associated with the restriction of phagosome damage (Fig. 5D and fig. S4E). Confirming a role in restriction of damage, an obvious increase in GAL8+ damaged Mtb phagosomes was observed in *ATG16L1* KO and *ATG7* KO macrophages (Fig. 7, A and B, and fig. S5A). We also observed an increase in GAL3+ damaged Mtb phagosomes in *ATG16L1* KO macrophages (Fig. 7, A and C, and fig. S5A). The percentage of GAL3+ and GAL8+ Mtb phagosomes was not notably different between WT and *ATG13* or *FIP200* KO macrophages (Fig. 7, A to C, and fig. S5A). The unacidified status of multimembrane Mtb-LC3-TVS and increased phagosome damage under BAPTA-AM/BafA1 treatment and in *ATG16L1/ATG7* KO macrophage argued for a role for Atg8/LC3 lipidation in phagosome repair. Endosomal sorting complex required for transport (ESCRT)-III recruitment at the injured membrane site depends on the Ca^2+^ binding to apoptosis-linked gene-2 (ALG-2) and subsequent recruitment of ALIX, VPS4A, and the ESCRT-III machinery for repair (*43*, *44*). We analyzed the recruitment of the ESCRT-III component CHMP4B and GAL8 to the Mtb compartments and found that only 10.8% of GAL8+ were CHMP4B+ (fig. S5, B to D). CHMP4B was associated but did not completely colocalize with GAL8+ compartments (fig. S5B). Moreover, BafA1 treatment, which blocks Mtb-LC3-TVS formation, had no effect on the recruitment of CHMP4B (fig. S5E). BAPTA-AM only has a very minor effect on impairing the CHMP4B+ Mtb phagosome formation: A large population of CHMP4B+ GAL8− damaged phagosomes were observed under BAPTA-AM treatment (fig. S5, B to D). To further confirm the cross-talk between Mtb-LC3-TVS and ESCRT recruitment, we analyzed the recruitment of ALG-2 to damaged Mtb phagosomes under BAPTA-AM and BafA1 treatment. Similar to CHMP4B, ALG-2 was associated but did not completely colocalized with GAL8+ compartments (fig. S5F). BAPTA-AM treatment impaired ALG-2 recruitment, although a fraction of ALG-2+ GAL8− damaged phagosomes remained (fig. S5, F to H). Only 35% of the GAL8+ Mtb phagosomes were positive for ALG-2. BafA1 treatment had no effect on the recruitment of ALG-2 (fig. S5I). These results suggest that Ca^2+^ leakage–dependent Mtb-LC3-TVS formation is independent of the ESCRT membrane repair machinery.

To define the function of Ca^2+^ leakage–dependent LC3-TVS formation, we performed high-content live-cell imaging of WT, *ATG16L1* KO, *ATG7* KO, and *FIP200* KO macrophages infected with Mtb WT or Mtb ΔRD1 in the presence of propidium iodide (PI), a probe that detects cell death after a compromise in the plasma membrane (*31*). We observed an increase in Mtb WT replication in *ATG16L1* and *ATG7* KO macrophages whereas only a minor increase in Mtb ΔRD1 replication. In contrast, *FIP200* depletion had no effect on Mtb replication (Fig. 7, D and E, and fig. S6, A to C). KO of these genes has no effect on Mtb uptake (fig. S6D). Compared to WT macrophages and *FIP200* KO macrophages, there were more cell death in *ATG16L1* and *ATG7* KO macrophages when infected with Mtb WT (Fig. 7F). To verify the function of canonical autophagy for Mtb control in a more physiological relevant human macrophage cell model, we generated *FIP200* KO iPSDMs (fig. S6E). Whereas Mtb WT showed an increased growth in *ATG7* KO iPSDMs (*23*), we found that *FIP200* depletion had no effect on Mtb growth in iPSDMs (fig. S6, F to H). These results indicate that the Ca^2+^ leakage–dependent multimembrane LC3-TVS formation is an anti-Mtb cell autonomous response distinct from canonical autophagy.

## DISCUSSION

Despite compelling evidence showing Mtb Atg8ylation during infection of macrophages (*21*, *45*), the cellular mechanism by which this association with ATG8/LC3 targets Mtb to lysosomes in macrophages has remained elusive. This is due to the fact that both canonical and noncanonical pathways of Atg8ylation are reported to control intracellular Mtb (*2*). It has been widely believed that the effect of the canonical autophagy pathway in Mtb control, known as xenophagy, is by directing intracellular Mtb to degradation by fusing with lysosomes, but direct evidence of how this process terminate was lacking (*21*).

Here, we identified a Ca^2+^ leakage–dependent pathway implicated in the association of ATG8/LC3 to multiple membranes after damage that restrict Mtb phagosome damage, without directly targeting Mtb to the lysosomal degradation pathway (fig. S7). Using an autophagic flux probe that allows us to visualize lysosome-dependent ATG8/LC3+ compartment turnover, we observed that, even after long periods of infection, the Mtb LC3+ multimembrane does not target bacteria to lysosomes. It is widely believed that targeting intracellular bacteria to lysosomes will restrict or eventually kill them. However, there is limited evidence showing that this is the case and there is evidence that bacteria, including mycobacteria, can survive in acidic compartments (*46*), suggesting that multiple pathways contribute to the killing of intracellular bacteria or that bacteria counteracts acidification and lysosomal activity (*47*, *48*). There is less colocalization between LC3-positive *Salmonella* and zymosan phagosomes with late endocytic organelles (*49*, *50*). The formation of Mtb-LC3 positive membranes is closely associated with membrane damage (*25*), and these damage-related LC3+ compartments undergo minimal acidification (*51*, *52*).

This Ca^2+^ leakage–dependent pathway is mechanistically different from previously described conventional, canonical, and noncanonical autophagy pathways. Mtb phagosomes undergoing damage show a very rapid (within minutes) response to Ca^2+^ leakage. This is because a BAPTA-dependent and EGTA-independent process likely involves a fast and local Ca^2+^ rather than a steady-state Ca^2+^ levels. Ca^2+^ leakage has been implicated in many mechanisms of membrane repair, including not only the recruitment of the ESCRT-III machinery (*43*) but also ESCRT-independent pathways (*53*). Our data indicate that, in addition to membrane repair mechanisms, Ca^2+^ leakage triggers Atg8ylation not only on Mtb damaged phagosomes but also additional membranes recruited to the damaged phagosome. We propose that Ca^2+^ leakage is a conserved signal of phagosome damage recognition. As Ca^2+^ is present in all phagosomes at high levels, it is likely that this represents a common response to endomembrane damage. Previous research has shown that the autophagy machinery cooperates with the ESCRT complex during membrane damage to facilitate the repair or removal of damaged membranes (*54*, *55*). In vitro studies have demonstrated that LC3A and LC3C can bind to ESCRT components, suggesting a role in maintaining autophagosomal membranes in a sealed state (*56*). However, the recruitment of ESCRT components and ATG8/LC3 occurs in distinct spatial and temporal patterns in the *Dictyostelium discoideum*–*M. marinum* infection model (*57*). Our data suggest that the LC3-TVS relevant damage restriction mediated by Ca^2+^ leakage is ESCRT independent, and it remains to be defined how precisely Ca^2+^ locally regulates the Atg8ylation machinery.

Several studies have suggested intense membrane remodeling of damaged mycobacterial phagosomes (*23*, *25*, *57*). We observed that multiple membranes are Atg8ylated in response to Ca^2+^ leakage induced by damage, in a process that is different from previously described single- or double-membrane pathways. Our CLEM approach shows that Mtb phagosomes undergoing damage are surrounded by intricate membranes that are Atg8ylated, indicating that single or double ATG8/LC3+ membranes are rare or, eventually, a very transient initial step after damage. We observed two distinct phenotypes during Mtb-LC3-TVS formation in 48 hours of live-cell imaging: Mtb-LC3-TVS encapsulates Mtb and never undergoes acidification; Mtb segregated from these LC3-TVS, and the remaining LC3-TVS fused with acidic endolysosomes for degradation. The distinction between these two phenotypes may arise from varying levels of Mtb-induced phagosome damage and the dynamic balance of host cell membrane repair processes. Alternatively, the phenotypic differences could also reflect heterogeneity in the metabolic (or any other) activity of Mtb and membrane-disruptive capacity.

A recent study has proposed a link between lysosome damage and LC3 lipidation on single damaged lysosomal membranes (CASM) (*15*). However, the mechanism about how this process is triggered remains unclear. Further studies will help to understand whether Ca^2+^ leakage–dependent ATG8/LC3 membrane lipidation is also a general response to lysosome damage. Our live-cell single Mtb phagosome studies show that recruited acidic lysosomes likely contribute to complex membrane formation in a process that does not involve single membranes (e.g., phagosomes). This mechanism could be related to a previous study showing that early endocytic vesicles contribute to Mtb phagosome integrity in mouse macrophages (*58*). In this context, the autophagy machinery has been suggested to contribute to maintain membrane integrity during bacterial infection such as the *Salmonella*-containing phagosome membrane (*59*), *M. marinum*–containing phagosome membrane in the *D. discoideum* model (*57*), and cell membrane integrity during *Listeria monocytogenes* cell-to-cell spread (*60*). Our data indicate that the Ca^2+^ leakage–dependent LC3-TVS formation is different from the one observed in *Salmonella* or *Listeria* as autophagy initiation seems to be partially required for *Salmonella* phagosome integrity (*59*), and ATG16L1-dependent pathway promotes plasma membrane repair during *Listeria* spread is LC3 lipidation independent (*60*). Our live-cell single-organelle imaging data indicate that the main function of the Ca^2+^ leakage–dependent Atg8ylation is to locally contain and restrict the damage of Mtb phagosomes. Although it remains to be defined whether Atg8ylation is directly involved in membrane repair, our results suggest it can potentially contribute to membrane fusion with lysosomes and membrane remodeling. In addition to membrane repair, LC3-TVS formation could represent a membrane accumulation process driven by continuous damage, with these accumulated membranes potentially playing a role in restricting Mtb growth. Further studies will define the function of these multimembrane compartments.

The Ca^2+^ leakage–dependent ATG8/LC3 membrane lipidation requires the Atg8ylation machinery, but it is independent of canonical autophagy initiation as it has been described for other intracellular pathogens (*61*, *62*). In the context of tuberculosis, these specific ATG-dependent cellular mechanisms could help to better understand and interpret in vivo infection experiments showing the requirement of specific Atg genes in different systems (*22*, *63*). The Ca^2+^ leakage–dependent ATG8/LC3 membrane lipidation is independent of canonical autophagy components and depends on membrane damage as it has been described for other processes such as LAP or CASM. However, this mechanism involves multiple membranes and does not target Mtb to phagolysosomes. Recent studies reported that Ca^2+^ leakage induces sphingomyelin scrambling, which triggers the recruitment of the ATG12-ATG5-TECPR1 E3-like complex and LC3 lipidation in response to membrane damage (*61*, *62*, *64*). These findings suggest the presence of alternative LC3 lipidation mechanisms and the involvement of distinct ATG8 family members in membrane damage responses. These mechanisms may play a role during Mtb phagosome damage, although further investigation is required to confirm this. The mechanism of Mtb-LC3-TVS membrane lipidation requires the V-ATPase/Atg16L1 complex but different from other systems as it only requires a functional V-ATPase but not changes in proton content of the Mtb phagosome lumen, differentiating this Ca^2+^ leakage–dependent pathway from other processes previously described in viral infections (*65*).

Studies in fixed cells showed that both Gal3 and Gal8 had been associated to Mtb phagosomes undergoing damage (*58*, *66*). Our spatiotemporal analysis provides further evidence that GAL3 is not a “universal” marker of damage (*67*) as GAL3 only labels very late steps of damaged Mtb phagosomes. Our data indicate that Ca^2+^ leakage–dependent GAL8 recruitment is a more reliable marker of damage as it labels both early and late stages of membrane damage. These results highlight that other markers need to be considered when studying membrane damage, particularly in the context of limited versus extensive damage. The general idea is that Galectins recognize carbohydrate moieties present in the luminal side of membranes undergoing damage. However, numerous glycoproteins are also present in the lumen of endolysosomes. Our data provide evidence that Galectins recognize specific phagosome components that are exposed in a spatiotemporally regulated manner and partially controlled by Ca^2+^ leakage.

On the basis of the data presented here, we propose that Ca^2+^ leakage–dependent Atg8ylation-mediated repair is a local response of the macrophage to restrict damage and Mtb replication. It is likely that this is a conserved pathway in response to membrane damage, and further studies will define whether other intracellular pathogens that induce Ca^2+^ leakage trigger similar responses in host cells during infection or developed strategies to subvert it.

### Limitations

Because of variations in the timing of Mtb entry into cells and subsequent phagosome damage, as well as the limitations of high-resolution live-cell imaging in tracing only a limited number of cells and channels, defining the accurate zero time point for phagocytosis while having a robust quantitative analysis is challenging. In this study, time zero in the series represents the time point for this sequence start but not the time point of phagocytosis. For Mtb tracking in the long-term live-cell imaging in this study, we used TrackMate, which allows us to efficiently track Mtb movement and quantify signal intensities over time. However, TrackMate does not directly segment cells or bacteria but creates fixed-radius spheres around detected centroids. Although this method can sometimes include signals beyond the vicinity of Mtb, it does not compromise the validity of our results. The GCaMP6f Ca^2+^ probe has certain inherent limitations: Detecting Ca^2+^ signals rely on real-time fluorescence intensity changes, and the amplitude of these changes varies between individual events, as demonstrated in Fig. 1C. This variability likely depends on factors such as the starting point of the imaging (defining the baseline), the dynamic interaction between membrane damage and repair, as well as the extent of the damage.

## MATERIALS AND METHODS

### Cells

THP-1 cells (European Collection of Authenticated Cell Cultures, no. 88081201) were cultured in RPMI medium (RPMI 1640 medium with GlutaMAX supplement, Thermo Fisher Scientific, 72400047) supplemented with 10% fetal bovine serum (FBS; Thermo Fisher Scientific, A52094) at 37°C with 5% CO_2_ in a humidified incubator. For THP-1 macrophage differentiation, THP-1 monocytes were plated and cultured in growth medium (RPMI with 10% FBS) with 100 nM phorbol 12-myristate 13-acetate (PMA; Sigma-Aldrich, 16561-29-8) for 24 hours. After removing PMA, THP-1 macrophages were cultured in growth medium for 48 hours and then applied for following experiments. Cells were authenticated with short tandem repeats (STR) profiling and checked for mycoplasma contamination monthly. For Torin-1 and BafA1 treatment in autophagic activity assays, after plating and differentiation, THP-1 macrophages were cultured in growth medium supplemented with 1 mM Torin-1 [Cell Signaling Technology (CST), 14379S] for 2 hours or with 100 nM BafA1 (Sigma-Aldrich, B1793-10UG) for 4 hours.

### Human iPSC culture and iPSDM differentiation

Human iPSC and iPSDM cultures were established using KOLF2 iPSCs sourced from Public Health England Culture Collections (catalog number 77650100). KOLF2 iPSCs were cultured in Vitronectin XF (StemCell Technologies, 100-0763) coated plates with Essential 8 (E8) medium (Thermo Fisher Scientific, A1517001). The cells were authenticated by STR profiling upon receipt and monitored monthly for mycoplasma contamination. Passaging was performed at a ratio of 1:6 once the cells reached 70% confluency using Versene (Thermo Fisher Scientific, 15040066). Monocyte factories were established following a previously reported protocol. Briefly, a single-cell suspension of iPSCs was produced with TryplE (Thermo Fisher Scientific, 12604013) and seeded into AggreWell 800 plates (StemCell Technologies) with 4 × 10^6^ cells per well. The forming embryonic bodies (EBs) were then fed daily with E8 medium supplemented with human BMP4 (50 ng/ml; PeproTech, 120-05), human vascular endothelial growth factor (50 ng/ml; PeproTech, 100-20), and human stem cell factor (20 ng/ml; PeproTech, 300-07) for 3 days. On day 4, the EBs were collected, filtered, and seeded 250 to 300 per T225 flask in factory medium. Factory medium consisted of X-VIVO15 (Lonza, LZBE02-061Q) supplemented with GlutaMAX (Thermo Fisher Scientific, 35050061), 50 μM β-mercaptoethanol (Thermo Fisher Scientific, 31350010), human macrophage colony-stimulating factor (M-CSF) (100 ng/ml; PeproTech, 300-25), and human interleukin-3 (25 ng/ml; PeproTech, 200-03). Monocyte factories were fed weekly with factory medium for 5 weeks until monocytes were observed in the supernatant. Up to 50% of the supernatant was collected weekly and centrifuged, and the cells were resuspended in X-VIVO15 supplemented with GlutaMAX and human M-CSF (100 ng/ml). The resuspended monocytes were plated at 4 × 10^6^ cells per 10-cm petri dish to differentiate over 7 days. On day 4, a 50% medium change was performed. To detach iPSDMs, plates were washed with phosphate-buffered saline (PBS), incubated with Versene for 15 min at 37°C and 5% CO_2_, diluted 1:3 with PBS, and gently scraped. Macrophages were centrifuged and plated for experiments in X-VIVO15 medium.

### Lentivirus production and transfection

Target sequences were polymerase chain reaction (PCR) amplified from cDNA libraries or existing plasmids and cloned into a lentivirus transfer plasmid with EF1a promoter, which was kindly provided by M. Strom (The Francis Crick Institute). Lentiviral production was performed in 293FT cells using third-generation packaging system with three packaging plasmids (pLP1, pLP2, and pLP/VSVG) and a transfer plasmid containing target gene for expression or guide RNA (gRNA) for KO cell construction. Virus-containing supernatant was harvested 2 and 3 days posttransfection and filtered through 0.45-μm PES membrane filters. The lentivirus was concentrated with Lenti-X Concentrator (Takara, 631232), and the titer was defined by quantitative reverse transcription PCR. THP-1 monocytes were counted and incubated with growth medium containing lentivirus [multiplicity of infection (MOI): 1] and polybrene (10 mg/ml; Merck, TR-1003-G) for 72 hours. For fluorescent-tagged protein expression, the THP-1 monocytes with target gene expression were selected by fluorescence-activated cell sorting (FACS) and then back to culture with growth medium. A suitable number of cells were collected later and subjected to the following experiments. KOLF2 iPSCs were incubated with growth medium containing lentivirus (MOI: 0.5) and polybrene (10 mg/ml; Merck, TR-1003-G) for 72 hours. Fluorescent-tagged protein expressed iPSCs were sorted by FACS and then back to culture and iPSDM differentiation.

### Generation of CRISPR-Cas9 KO THP-1 cells

The gRNAs for target gene were cloned into the LentiGuide-Puro vector (Addgene, 52962). The lentiviruses were produced with LentiCas9-Blast plasmid (Addgene, 52963) and LentiGuide-gRNA-Puro plasmid. THP-1 monocytes were then infected with lentivirus for Cas9 expression (MOI: 1) and gRNA expression (MOI: 0.5) at the same time. After 72-hour infection, the THP-1 monocytes were incubated with growth medium containing puromycin (2 mg/ml) and blasticidin (15 mg/ml) for 7 days. Live cells were single-cell sorted into 96-well plates containing growth medium. DNAs from single-cell clones were extracted and sequenced to analyze CRISPR targeting by ICE Analysis (Synthego). Target clones were then confirmed by Western blot. After gene type and protein level confirmation, two to five KO clones for each of the gene underwent key phenotype confirmation.

The gRNAs used for generating KO THP-1 cells in this study are as follows (5′-3′): FIP200 gRNA-1: TATGTATTTCTGGTTAACAC, FIP200 gRNA-2: TCTTCTAGTAACTGTATCAG, ATG7 gRNA-1: CCTCATAGGTGGACCACAGG, ATG7 gRNA-2: GCTGCCAGCTCGCTTAACAT, ATG13 gRNA-1: CTGTCCCAACACGAACTGTC, ATG13 gRNA-2: ACTTGGCATTCATGTCTACC, ATG16L1 gRNA-1: TGAATTACACAAGAAACGT, and ATG16L1 gRNA-2: TGGTGCTTAATCCTCAGTT.

### Generation of CRISPR-Cas9 KO human iPSCs

Nucleofection of KOLF2 iPSCs for gRNA and S.p.Cas9 delivery was performed using the Amaxa 4D-Nucleofector (V4XP-3024, Lonza). For each nucleofection, 1 × 10^6^ human iPSCs were resuspended in 100 μl of P3 buffer (Lonza, V4XP-3024) containing 20 μg of S.p.Cas9 (Alt-R S.p. Cas9 Nuclease V3, 1081059, IDT) and 16 μg of synthetic, chemically modified single gRNAs (Synthego). The cell suspension and Cas9-RNP mixture were nucleofected using program CA-137. Following nucleofection, single clones were manually picked and subsequently analyzed by sequencing and Western blotting. The gRNAs used for generating FIP200 KO iPSCs are as follows (5′-3′): FIP200 gRNA-1: CAAGATTGCTATTCAACACC and FIP200 gRNA-2: TGCTTTGAATGGCATGCTTA.

### Mtb culture and infection

The preparation of fluorescent Mtb, as well as its culture and infection protocols, has been described previously (*68*). In brief, WT Mtb H37Rv and Mtb H37Rv ΔRD1 strains were provided by D. Young and S. Hingley-Wilson. E2-Crimson Mtb was generated by transformation with pTEC19 (Addgene, 30178, deposited by L. Ramakrishnan). Mtb strains were verified by sequencing and tested for phthiocerol dimycocerosate positivity by thin-layer chromatography of lipid extracts. Mtb strains were cultured in Middlebrook 7H9 supplemented with 0.2% glycerol, 0.05% Tween 80, and 10% albumin dextrose catalase.

For macrophage infections, Mtb was grown to an optical density (OD_600_) of ~0.8, then centrifuged, and washed twice with PBS. The pellet was shaken with 2.5- to 3.5-mm glass beads for 2 min to produce a single-cell suspension. Bacteria were resuspended in cell culture medium, and the OD600 was determined. Bacteria were diluted to the appropriate OD for the required MOI before being added to cells (assuming OD_600_ = 1 equates to 10^8^ bacteria/ml). For all the experiments apart from live-cell imaging of Mtb replication experiments in this study, we used an MOI of 2 to ensure sufficient infection levels for robust analysis. After 2-hour uptake, cells were washed with PBS to remove the extracellular bacteria and then change to normal medium. For chemical treatment during infection, after Mtb uptake and washing with PBS, cells were incubated with medium containing 100 nM BafA1, 1 μM Torin-1, 50 μM CQ (Sigma-Aldrich, C6628), 50 μM BAPTA-AM (Abcam, ab120503), or 50 μM EGTA-AM (Thermo Fisher Scientific, E1219). Cells were then incubated for appropriate time points before collection for analysis.

### Immunofluorescence of macrophages

Cells were fixed in 4% paraformaldehyde (PFA) in PBS, followed by quenching with 50 mM NH_4_Cl in PBS for 10 min. Next, cells were permeabilized with 0.05% saponin dissolved in 0.1% bovine serum albumin (BSA) in PBS for 20 min. After permeabilization, cells were blocked with 0.1% BSA in PBS for 10 min. The primary antibody was diluted 1:200 in blocking buffer and incubated with the cells for 1 hour at room temperature or overnight at 4°C. Following primary antibody incubation, cells were washed three times for 10 min each with PBS. Subsequently, the secondary antibody in blocking buffer was incubated with the cells for 1 hour at room temperature or overnight at 4°C. After secondary antibody incubation, cells were washed three times for 10 min each with PBS. Nuclei were stained with 4′,6-diamidino-2-phenylindole (DAPI) (Thermo Fisher Scientific, D1306) for 10 min followed by a 10-min washing with PBS. For mounting, coverslips were mounted with DAKO mounting medium (DAKO, S3023), or cells were mounted with PBS in a 96-well plate or 18-well plate. The antibodies used are as follows: GAL3 (BioLegend, 125410), GAL8 (R&D Systems, AF1305), LC3B (Sigma-Aldrich, L7543), p62 (CST, 88588), ATP6V1D (Abcam, ab157458), and CD63 (Abcam, ab217345).

### Western blot analysis

Macrophages were washed once with PBS and then lysed for 20 min on ice in radioimmunoprecipitation assay buffer (Millipore, 20-188) with protease inhibitor (Thermo Fisher Scientific, 78445). After centrifugation at 13,000 rpm for 15 min at 4°C, the supernatants were boiled at 100°C for 10 min in LDS sample buffer (Thermo Fisher Scientific, NP008) supplemented with NuPage Sample Reducing Agent (Thermo Fisher, NP009). Subsequently, the samples were loaded into a 4 to 12% Bis-Tris gel (Thermo Fisher Scientific, WG1403BOX) and underwent electrophoresis. The separated proteins were then transferred onto a polyvinylidene difluoride membrane using an iBlot2 (Thermo Fisher Scientific, IB21001). Following transfer, the membranes were blocked in 5% skimmed milk powder in tris-buffered saline (TBS) with 0.05% Tween 20 (TBS-T) for 1 hour at room temperature. Next, the membranes were incubated with primary antibodies for 1 hour at room temperature or overnight at 4°C. After washing three times in TBS-T, the membrane was incubated with horseradish peroxidase (HRP)–conjugated secondary antibodies for 1 hour at room temperature. Signals on the membrane were imaged on an Amersham GE Imager 680 (GE Healthcare) with chemiluminescence reagent (Bio-Rad). The antibodies used are as follows: p62 (CST, 5114), ATG7 (CST, 8558), ATG16L1 (CST, 5504S), β-actin–HRP (CST, 12262), LC3B (CST, 2775), FIP200 (Proteintech, 17250-1-AP), and ATG13 (Sigma-Aldrich, SAB4200100).

### Imaging for fixed samples

Coverslips or glass-bottom dishes were imaged using a Leica STELLARIS 5 inverted confocal microscope (Leica Microsystems) with a 63× 1.4–numerical aperture (NA) oil immersion objective. Fluorescence was detected using Leica HyD detectors. Laser and detector settings were kept constant for each biological replicate of experiments.

### Long-term live-cell imaging during Mtb infection

A total of 1 million macrophages were differentiated and growth on 35 mm glass-bottom dish (Willco, GWST-3512; ibidi, 81158). After 2 hours of Mtb uptake, the dishes were sealed with Parafilm, loaded into a holder, and imaged on a confocal microscope (Leica STELLARIS 5) equipped with an environmental chamber (Okolab) set at 37°C with 5% CO_2_ within a biosafety level 3 (BSL3) environment. Samples were imaged using a 63× 1.4-NA oil immersion objective. For live-cell imaging with LTR, GFP-LC3B stably expressing THP-1 macrophages were infected with E2-Crimson Mtb. After uptake and washing, cells were cultured and imaged with growth medium containing 100 nM LysoTracker Red DND-99 (Thermo Fisher Scientific, L7528). For live-cell imaging under EGTA-AM/BAPTA-AM treatment, after Mtb uptake and washing, cells were cultured and imaged with growth medium containing 50 μM BAPTA-AM (Abcam, ab120503) or 50 μM EGTA-AM (Thermo Fisher Scientific, E1219).

### High-content live-cell imaging of Mtb replication

A total of 90,000 THP-1 macrophages were seeded per well on olefin-bottom 96-well plates. Cells were incubated with Mtb (MOI: 0.2) for 2 hours. After Mtb uptake, cells were washed with PBS and replaced with growth medium containing PI (0.4 μg/ml; Abcam, ab14083). Imaging was performed using an OPERA Phenix microscope (PerkinElmer) with a 40× 1.1-NA water-immersion objective in BSL3. Four planes with a 1-μm distance covering 20 fields of view were monitored over time, and snapshots were taken every 2.5 hours for 72 hours. For assessing bacterial replication and cell death, analysis was performed using Harmony 4.9 software. Maximum projection of individual *z*-planes with an approximate distance of 1 μm was used. To perform cellular segmentation, the “Find Texture Regions” building block was trained in the bright-field channel to segment cellular areas. Following cellular segmentation, the “Find Spots” and “Find Nuclei” building blocks were used to segment Mtb and PI-positive nuclei. To determine the bacteria area and PI-positive nuclei over time, the spot area and number of PI-positive nuclei were summed for each time point. The fold change calculation is normalized to the initial time point (time 0), defined as the frame immediately after a 2-hour uptake period, and reflects the relative intracellular area occupied by Mtb. This normalization method allows us to accurately track changes in Mtb growth over time from a consistent baseline. Mtb growth as fold change was calculated using the formula: (sum of intracellular Mtb area for each time point)/(sum of intracellular Mtb area at *t*_0_ hour). In the live-cell imaging of Mtb replication experiment in this study, we used a lower MOI of 0.2. This choice was made to better mimic physiological conditions and to minimize the formation of large Mtb clumps, which could interfere with the final analysis. Because Mtb replication in this experiment is quantified based on the intracellular area occupied by Mtb, we found that a lower MOI reduced the risk of clump formation and provided a more accurate measurement of Mtb growth over time.

### CLEM sample preparation

A total of 10^6^ macrophages were differentiated and grown on 35-mm gridded MatTek dishes with a no. 1.5 coverslip (MatTek Corporation). After Mtb infection, samples were fixed by adding a mixture of 8% PFA in 200 mM Hepes (pH 7.4) buffer to culture medium (v/v) and incubated at room temperature for 15 min and then replaced with 4% PFA in 100 mM Hepes overnight at 4°C before imaging by confocal microscopy. After staining with DAPI in PBS for 20 min, samples were first imaged by a laser scanning confocal microscope (Leica STELLARIS 5) and then changed to 1% glutaraldehyde in 200 mM Hepes (pH 7.4) for 30 min at room temperature. Fluorescently imaged samples were processed for CLEM in a Biowave Pro (Pelco, USA) with use of microwave energy and vacuum. Cells were twice washed in Hepes (Sigma-Aldrich, H0887) at 250 W for 40 s and postfixed using a mixture of 2% osmium tetroxide (Taab O011) and 1.5% potassium ferricyanide (Taab, P018) (v/v) at an equal ratio for 14 min at 100-W power [with/without vacuum, 20 inches (67,727.8 Pa) Hg at 2-min intervals]. Samples were washed with distilled water twice on the bench and twice again in the Biowave 250 W for 40 s. Samples were stained with 1% aqueous uranyl acetate (Agar Scientific AGR1260A) in distilled water (w/v) for 14 min at 100-W power [with/without vacuum, 20 inches (67,727.8 Pa) Hg at 2-min intervals] and then washed using the same settings as before. Samples were dehydrated using a stepwise acetone series of 50, 75, 90, and 100% and then washed 4x in absolute acetone at 250 W for 40 s per step. Samples were infiltrated with a dilution series of 25, 50, 75, and 100% Ultra Bed Low Viscosity Epoxy Kit (EMS, 14310) (v/v) resin to propylene oxide. Each step was for 3 min at 250-W power [with/without vacuum, 20 inches (67,727.8 Pa) Hg at 30-s intervals]. Samples were then cured for a minimum of 48 hours at 60°C.

### Sample trimming and scanning electron microscopy array tomography acquisition

Referring to grid coordinates, the sample block was trimmed, coarsely by a razor blade and then finely trimmed using a 35° ultrasonic, oscillating diamond knife (DiATOME, Switzerland) set at a cutting speed of 0.6 mm/s, a frequency set by automatic mode, and a voltage of 6.0 V, on an ultramicrotome EM UC7 (Leica Microsystems, Germany) to remove all excess resin surrounding the region of interest. Serial sections were collected on ITO (indium tin oxide) glass slides for imaging using a Zeiss Gemini SEM 460.

### CLEM image alignment

Fluorescence .LIF files were converted to .tif file format, and linear adjustments made to brightness and contrast using FIJI (version 2.14.0/1.54t). Fluorescence images were aligned to serialEM micrographs (TrakEM2) using the BigWarp_fiji_7.0.7 plugin. No less than 10 independent fiducials were chosen per alignment for 3D image registration. When the fiducial registration error was greater than the predicted registration error, a nonrigid transformation (a nonlinear transformation based on spline interpolation, after an initial rigid transformation) was applied.

### Mtb tracking and live-cell imaging analysis

For segmenting and tracking of Mtb phagosomes during live-cell imaging, the “TrackMate” plugin of FIJI (version 2.9.0/1.53t) was used (*69*). Mtb were identified and tracked based on E2-Crimson fluorescence. The area surrounding Mtb was delineated by a circle with a similar diameter as the target Mtb phagosome. The background area refers to the portion of the cell without Mtb. The mean gray value of the fluorescence signals from the surrounding markers (such as LAMP1-GCaMP6f, RFP-GAL3, GFP-GAL8, RFP-GAL8, LTR, RFP-LC3, GFP-LC3, or mCherry-LC3) was traced and calculated. For the measurement of Mtb phagosomal Ca^2+^ release, 3- to 5-μm *Z*-stacks of each frame were acquired at 1024 × 1024 pixels, with a frame rate of 10 to 30 s per frame (varies depending on whether the single LAMP1-GCaMP6f signal or imaging with other markers simultaneously). For data analysis, regions with intensity transitions of LAMP1-GCaMP6f–positive Mtb phagosomes were manually cropped. The fluorescence intensity [in arbitrary units (a.u.)]–time curves were normalized by the intensity of the first image. The LAMP1-GCaMP6f signal trace was further normalized by the averaged initial baseline fluorescence of LAMP1-GCaMP6f surrounding Mtb, denoted as *F*_0_. The fluorescence of LAMP1-GCaMP6f surrounding Mtb at each time point was represented as *F*, and the dynamic change was calculated as *F*/*F*_0_. For parametric quantification, Ca^2+^ transient amplitude was analyzed, where the baseline fluorescence (*F*_0_) was subtracted from the peak fluorescence (*F*_p_) and then normalized by the baseline value (*F*_0_) to give the transient amplitude Δ*F*/*F*_0_ = (*F*_p_ − *F*_0_)/*F*_0_. For the measurement of intensity of other fluorescent markers, the fluorescence intensity [in arbitrary units (a.u.)] trace of the indicated marker was also normalized by the averaged initial baseline fluorescence surrounding Mtb, denoted as *F*_0_. The fluorescence of each marker surrounding Mtb at each time point was represented as *F*, and the dynamic change was calculated as *F*/*F*_0_.

### Image analysis and statistical analysis

To quantify Mtb GAL3/GAL8/ATG16L1/ATP6V1D compartments under different treatments, Mtb were identified based on E2-Crimson fluorescence. The area surrounding Mtb was delineated by a circle with a diameter similar to that of the target Mtb phagosome. Infected cells were defined as cells with Mtb localized within the cell cytoplasm. To accurately depict the variance in recruitment, the threshold intensity for defining positive structures was established as the average intensity of the fluorescence signals surrounding Mtb in control cells, where the Mtb phagosome has the indicated marker recruitment. Mtb structures with fluorescence intensity above this threshold were considered positive. For double-positive structures (e.g., LC3+ and Gal8+), the same threshold-based approach was applied to assess colocalization.

The sample size (with *n* representing the number of infected cells) and *P* values are reported in the figure legends. Each experiment was conducted with a minimum of three biological replicates. Graph plots and *P* values were generated using GraphPad Prism 10 software. Statistical comparisons were made using Two-tailed unpaired Student’s t tests. All data are presented as means ± SEM. Significance levels are denoted as follows: *P < 0.05; **P < 0.01; ***P < 0.001; and “n.s.” indicates no significant difference. All the error bars in the quantification graph are typically shown both above and below the data points. However, to more clearly display the data distribution, if the error bar extends below “0” and there is no data below 0, the error bar for that dataset will only be shown above the data points.
