# Supplementary material for: Mycobacterium tuberculosis phagosome Ca2+ leakage triggers multimembrane ATG8/LC3 lipidation to restrict damage in human macrophages
Source: Sci Adv. 2025 Mar 26;11(13):eadt3311. doi: 10.1126/sciadv.adt3311 (PMC11939036; doi:10.1126/sciadv.adt3311)
Supplement: Supplementary file 1 — Figs. S1 to S7 Legends for movies S1 to S6 [file sciadv.adt3311_sm.pdf]

Science Advances
AAAS

Supplementary Materials for

Mycobacterium tuberculosis phagosome Ca^{2+} leakage triggers multimembrane ATG8/LC3 lipidation to restrict damage in human macrophages

Di Chen *et al.*

Corresponding author: Di Chen, di.chen@crick.ac.uk; Maximiliano G. Gutierrez, max.g@crick.ac.uk

Sci. Adv. **11**, eadt3311 (2025)
DOI: 10.1126/sciadv.adt3311

The PDF file includes:

Figs. S1 to S7
Legends for movies S1 to S6

Other Supplementary Material for this manuscript includes the following:

Movies S1 to S6

Supplementary Figure 1

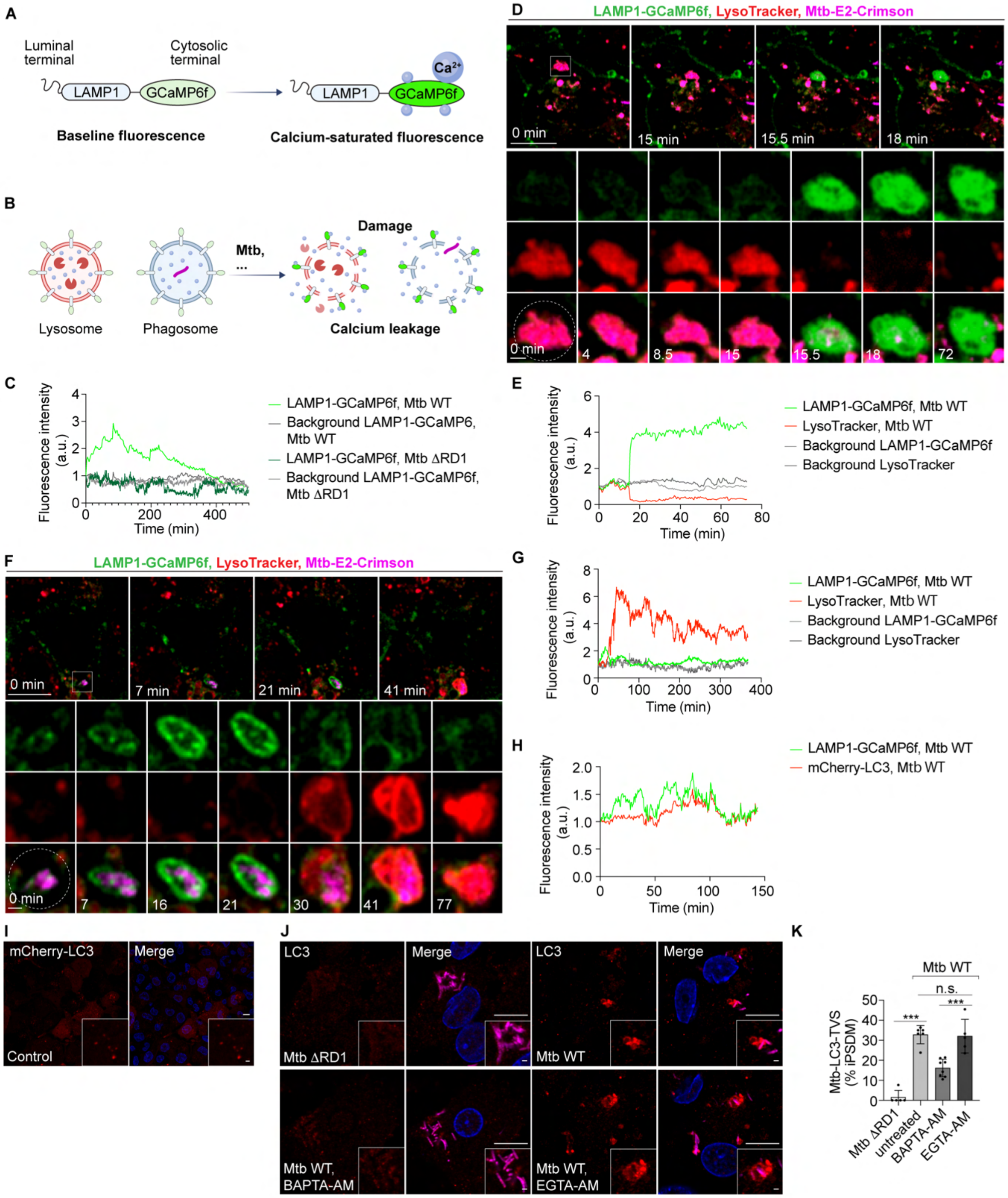

Figure S1: Ca^{2+} leakage during Mtb phagosome damage

A. The diagram shows the design of the LAMP-1-GCaMP6f reporter. The GCaMP6f is localized on the cytosolic side of human LAMP-1 and exhibits a baseline fluorescent intensity. When Ca^{2+}

binds to GCaMP6f, its fluorescent intensity significantly increases. **B.** The diagram shows the LAMP1-GCaMP6f localized to phagosomes exhibits a baseline fluorescent intensity. When Mtb induces phagosome damage, Ca^{2+} leaks out and binds to LAMP-1-GCaMP6f and resulting in a significant increase in fluorescent intensity. **C.** Shows the ratio change (F/F0) of LAMP1-GCaMP6f fluorescence intensity during Mtb WT and Mtb ΔRD1 infection as in **Figure 1A and 1B**. **D.** Live-cell imaging sequence showing phagosomal Ca^{2+} dynamic changes and LysoTracker Red signal during Mtb WT infection. White squares indicate the enlarged area. The dashed circle represents the Mtb area used for tracing the Mtb-phagosome in **S1E**. The images in **S1D** and **S1F** were processed with a Gaussian blur using a Sigma (radius) of 1. **E.** Ratio change (F/F0) of LAMP1-GCaMP6f and LysoTracker fluorescence intensity in **S1D**. **F.** Live-cell imaging sequence showing phagosomal Ca^{2+} dynamic changes and LysoTracker Red signal during Mtb WT infection. White squares indicate the enlarged area. The dashed circle represents the Mtb area used for tracing the Mtb-phagosome in **S1G**. **G.** Ratio change (F/F0) of LAMP1-GCaMP6f and LysoTracker fluorescence intensity in **S1F**. **H.** Ratio change (F/F0) of mCherry-LC3 and LAMP1-GCaMP6f fluorescence intensity surrounding Mtb in **Figure 1F**. **I.** THP-1 stably expressing mCherry-LC3B as a control of **Figure 1D**. **J.** LC3 staining in iPSDM at 4 hpi for Mtb WT and Mtb ΔRD1 infection (MOI:2) under indicated treatment. **K.** Quantification shows the percentage of infected iPSDM (4 hpi) showing Mtb-LC3 positive structures related to **S1J**. n (number of infected cells) =82-95, data points correspond to individual technical replicates from 3 independent experiments. **Scale bars**: 10 μm (main images), 1 μm (enlarged/inserted area).

Supplementary Figure 2

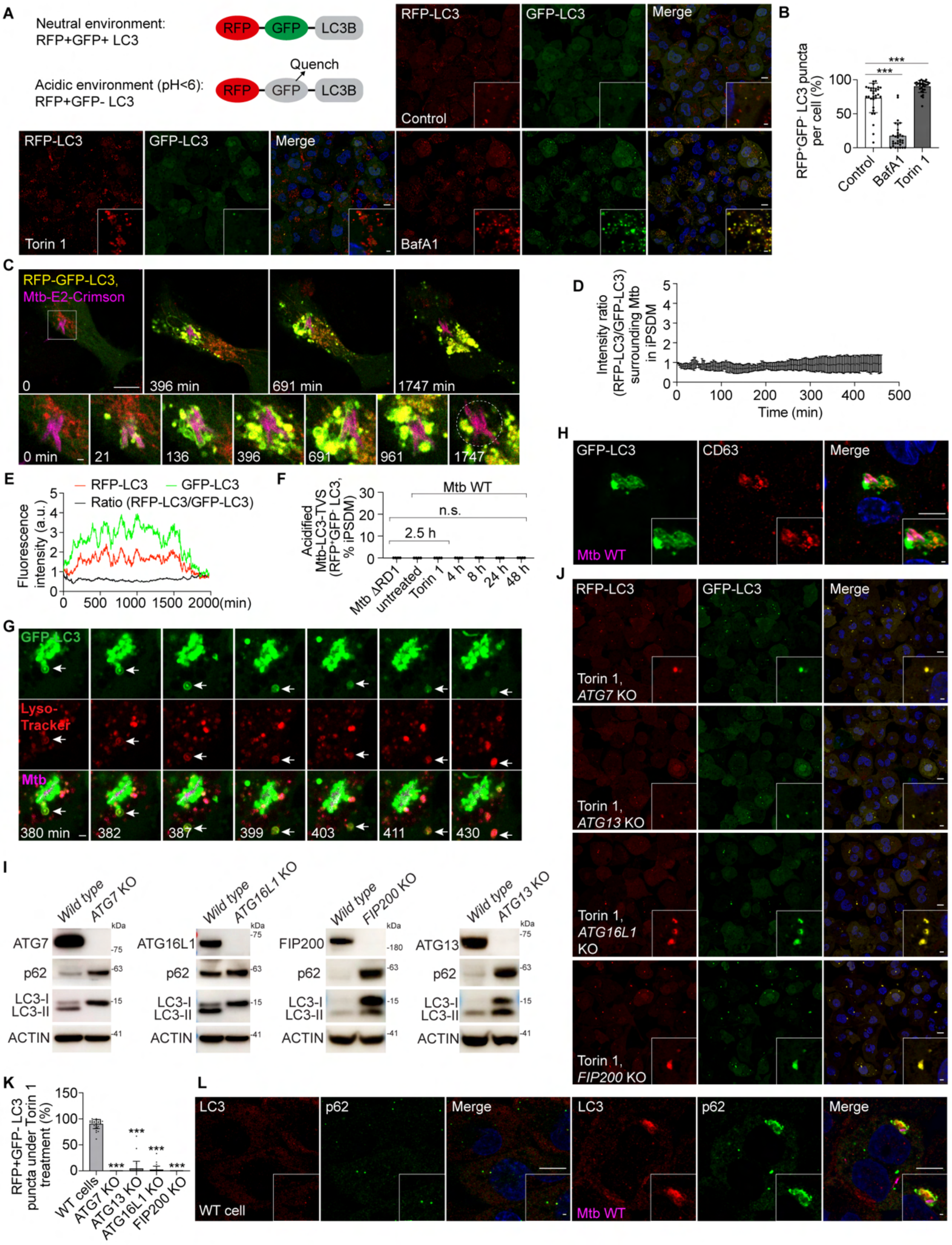

Figure S2: Mtb-LC3-TVS formation and ATG16L1-LC3 lipidation system

A. THP-1 macrophages stably expressing tandem RFP-GFP-LC3B under indicated treatment. The GFP signal is quenched in acidic lysosomes, $RFP^{+}GFP^{-}$LC3 indicates LC3 localized in the acidic compartment. **B.** Quantification shows the percentage of $RFP^{+}GFP^{-}$LC3 puncta per cell related to **S2A**. Data points correspond to individual cells=30 from 3 independent experiments. **C.** Live-cell imaging sequence showing RFP-GFP-LC3 changes during Mtb-WT infection in iPSDM. The square indicates the enlarged area. The circle of dashes represents the Mtb area used for tracing the LC3 signal in **S2E**. The Mtb escaped from 1721 min. **D.** The cumulative line scan ratio changes of RFP-LC3 (F/F0) to GFP-LC3 (F/F0) surrounding Mtb in iPSDM, with n=3. **E.** Shows the ratio change (F/F0) of RFP-LC3， GFP-LC3 and RFP-LC3(F/F0)/GFP-LC3(F/F0) in **S2C. F.** Quantification shows the percentage of infected iPSDM (MOI: 2) showing $RFP^{+}GFP^{-}$ Mtb-LC3-TVS. n (number of infected cells) =32-56, data points correspond to individual technical replicates from 3 independent experiments. **G.** Live-cell imaging sequence showing GFP-LC3 and LTR changes during Mtb-WT infection in series of **Figure 3B**. A LC3 positive vesicle segregated and then became LTR+ and gradually lost LC3 signal. **H.** CD63 staining in THP-1 macrophages stably expressing GFP-LC3B after Mtb WT infection (2.5 hpi, MOI:2). **I.** Western blot analysis of indicated THP-1 macrophages. **J.** Torin 1 treatment in RFP-GFP-LC3B stably expressing indicated THP-1 macrophages. **K.** Quantification shows the percentage of $RFP^{+}GFP^{-}$LC3 puncta per cell in indicated THP-1 macrophages related to **S2J**. The dataset for WT cell is same as **S2B**. For each condition, data points correspond to individual cells=30 from 3 independent experiments. **L.** p62 and LC3 staining in WT THP-1 macrophages uninfected or infected with Mtb WT for 2.5 h, as controls of **Figure 4C**. **Scale bars**: in **A**, **C, H, J, L**: 10 µm (main images), 1 µm (enlarged/inserted area), in **G**: 1 µm.

Supplementary Figure 3

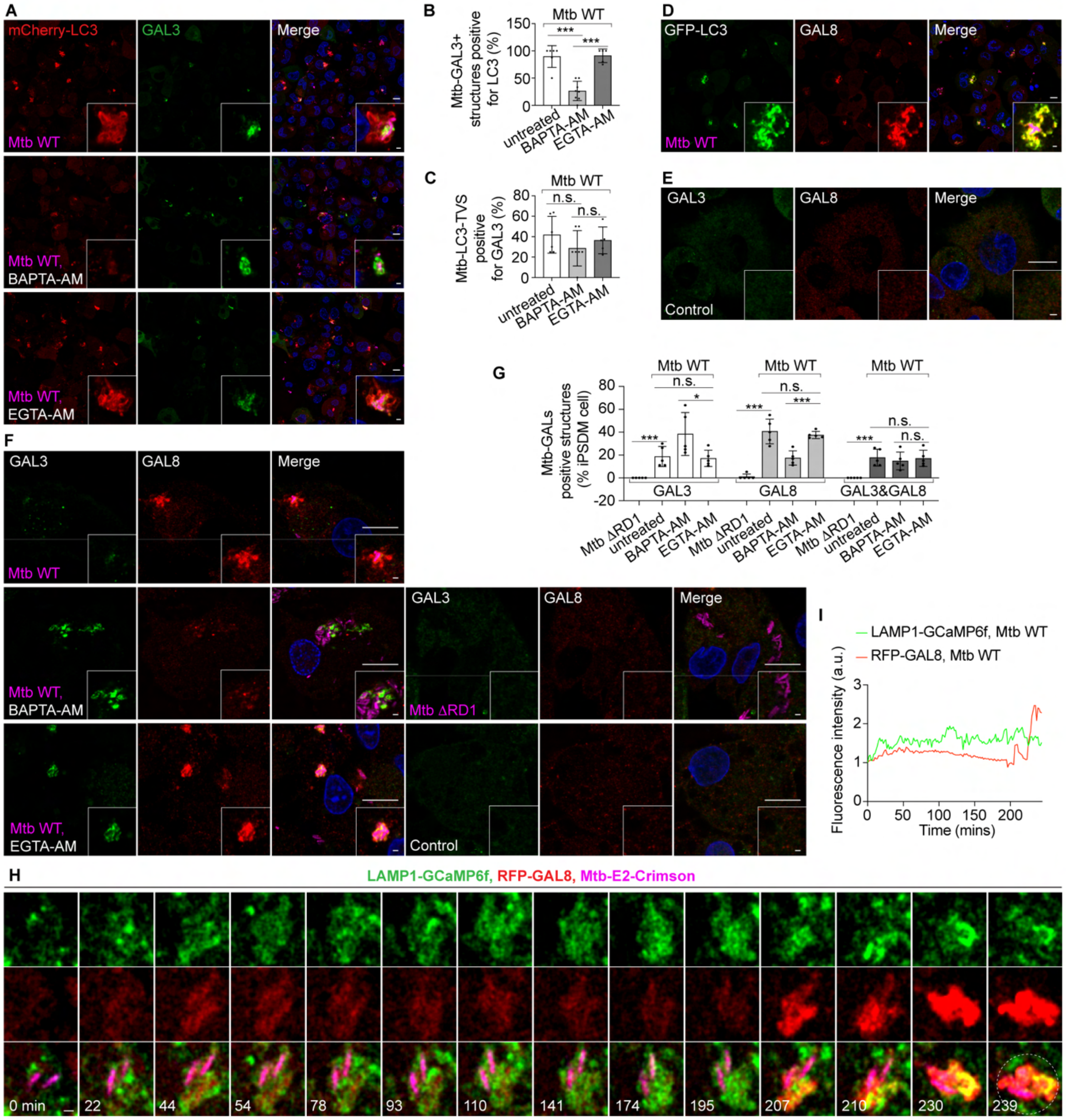

Figure S3: GAL8 is recruited to Mtb-LC3-TVS

A. GAL3 staining in THP-1 macrophages stably expressing mCherry-LC3B at 2.5 h hpi during Mtb WT and Mtb ΔRD1 infection (MOI:2) under indicated treatment. **B.** Quantification shows the percentage of Mtb-GAL3 positive structures positive for LC3 related to **Figure S3A**. n (number of infected cells) =118-163, data points correspond to individual technical replicates from 3 independent experiments. **C.** Quantification shows the percentage of Mtb-LC3-TVS positive for GAL3 related to **Figure S3A**. n (number of infected cells) =118-163, data points correspond to individual technical replicates from 3 independent experiments. **D.** GAL8 staining in THP-1

macrophages stably expressing GFP-LC3B after Mtb WT infection (2.5 hpi, MOI:2), as control of **Figure 5A**. **E.** GAL8 and GAL3 staining in THP-1 macrophages, as control of **Figure 5D**. **F.** GAL8 and GAL3 staining in iPSDM after Mtb WT and Mtb ΔRD1 infection (4 hpi, MOI:2) treated as indicated. **G.** Quantification shows the percentage of infected cells showing the Mtb-GAL3 positive, Mtb-GAL8 positive and Mtb-GAL3/GAL8 double positive structures in iPSDM, related to **S3F**. n (number of infected cells) =82-95, data points correspond to individual technical replicates from 3 independent experiments. **H.** Live-cell imaging sequence showing phagosomal Ca^{2+} dynamic changes and GAL8 recruitment during Mtb WT infection. The images in **S3H** were processed with a Gaussian blur using a Sigma (radius) of 1. The cycle of dashes represents the Mtb area used for tracing the Mtb-phagosome LAMP1-GCaMP6f and RFP-GAL8 signal in I. **I.** Shows the ratio change (F/F0) of Mtb WT-phagosomal LAMP1-GCaMP6f and RFP-GAL8 fluorescence intensity of MOI 1 in H. **Scale bars**: in **A, D, F**: 10 μm (main images), 1 μm (inserted area), in **H**: 1 μm.

Supplementary Figure 4

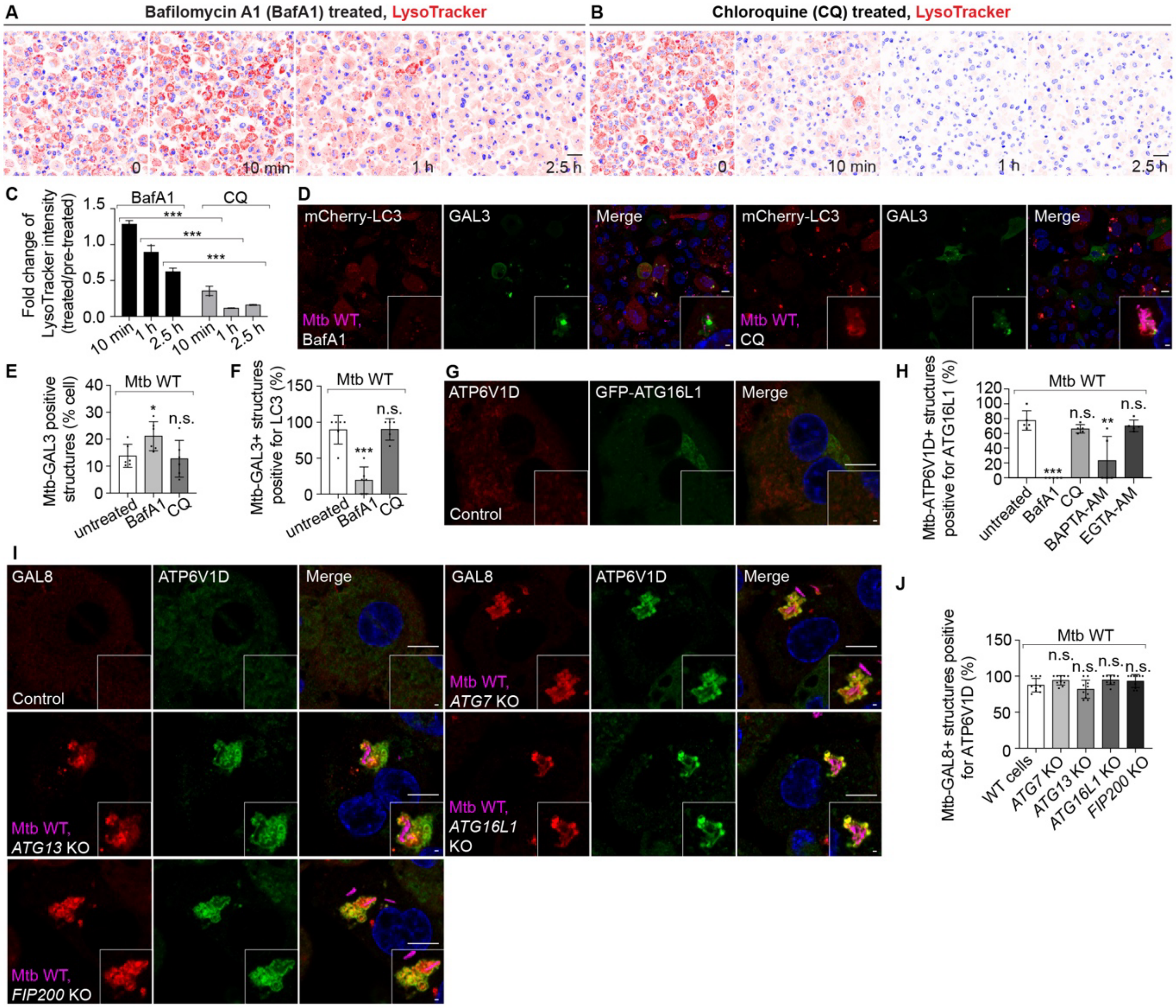

Figure S4: The recruitment of V-ATPase requires Ca^{2+} leakage but not LC3 lipidation

A. Live-cell imaging showing intensity changes of LysoTracker Red at indicated time point during Bafilomycin A1 (BafA1) treatment and Hoechst staining. **B.** Live-cell imaging showing intensity changes of LysoTracker Red at indicated time point during Chloroquine (CQ) treatment and Hoechst staining. **C.** Quantification shows the fold change in LysoTracker intensity at the indicated time points (10 min, 1 h, 2.5 h) during BafA1 or CQ treatment relative to the intensity before treatment (Time 0). n (number of cells) =596, 581, data points correspond to 3 individual focal plane. **D.** GAL3 staining in THP-1 macrophages stably expressing mCherry-LC3B infected with Mtb WT for 2.5 h (MOI:2) and treated with BafA1 or CQ. **E.** Quantification shows the percentage of infected cells showing Mtb-GAL3 positive structures related to **S4D**. n (number of infected cells) =128-163, data points correspond to individual technical replicates from 3 independent experiments. **F.** Quantification shows the percentage of Mtb-GAL3 positive structures positive for LC3 related to **S4D**. n (number of infected cells) =128-163, data points correspond to individual

technical replicates from 3 independent experiments. The data set for untreated cell is same as **Figure S3B**. **G.** ATP6V1D staining in uninfected THP-1 macrophages stably expressing GFP-ATG16L1, as a control of **Figure 6E**. **H.** Quantification shows the percentage of Mtb-ATP6V1D positive structures positive for ATG16L1 under indicated treatment, related to **Figure 6E**. n (number of infected cells) = 93-130, data points correspond to individual technical replicates from 3 independent experiments. **I.** GAL8 and ATP6V1D staining in *ATG7* KO, *ATG16L1* KO, *ATG13* KO, *FIP200* KO THP-1 macrophages infected with Mtb WT at 2.5 hpi. **J.** Quantification shows the percentage of Mtb-GAL8 positive structures positive for ATP6V1D in *ATG7* KO, *ATG16L1* KO, *ATG13* KO, *FIP200* KO THP-1 macrophages infected with Mtb WT at 2.5 hpi, related to **S4I**. n (number of infected cells) =165-240, data points correspond to individual technical replicates from 3 independent experiments. **Scale bars**: in **A, B**: 50 µm, in **D, G, I**: 10 µm (main images), 1 µm (inserted area).

Supplementary Figure 5

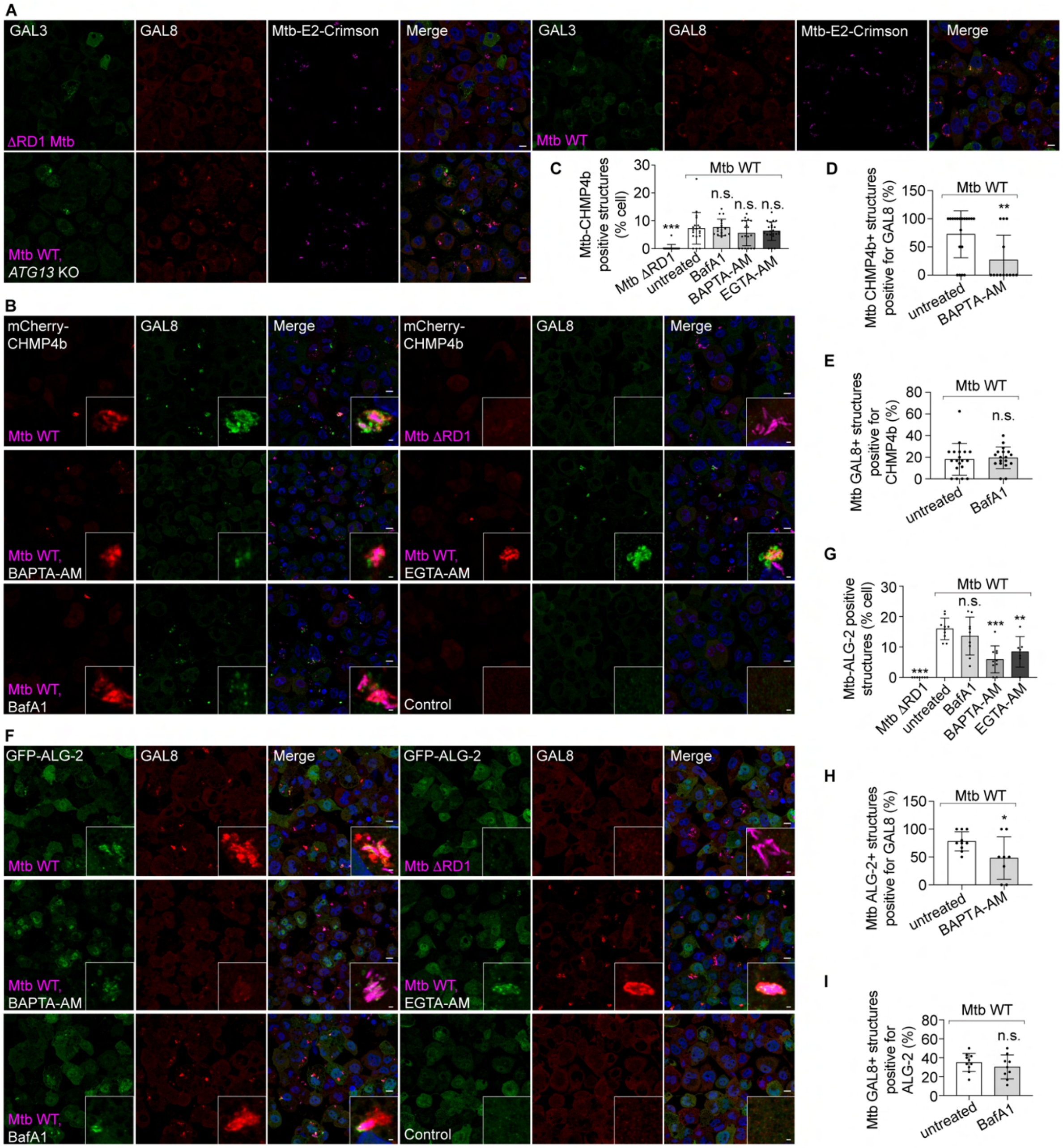

Figure S5: Mtb-LC3-TVS formation is independent of ESCRT recruitment

A. GAL8 and GAL3 staining in *ATG7* KO and *ATG13* KO THP-1 macrophages after Mtb WT and Mtb ΔRD1 infection in WT THP-1 macrophages (2.5 hpi, MOI:2). **B.** GAL8 staining in THP-1 macrophages stably expressing mCherry-CHMP4B infected or not with Mtb WT or Mtb ΔRD1 for 2.5 h (MOI:2) under the indicated treatments. **C.** Quantification shows the percentage of infected cells showing Mtb-CHMP4B positive structures under indicated treatment, related to **S5b**. n (number of infected cells) = 289-389, data points correspond to individual technical replicates

from 3 independent experiments **D.** Quantification shows the percentage of Mtb-CHMP4B positive structures positive for GAL8 under BAPTA-AM treatment, related to **S5B**. n (number of infected cells) = 346 and 289, data points correspond to individual technical replicates from 3 independent experiments. **E.** Quantification shows the percentage of Mtb-GAL8 positive structures positive for CHMP4B under BafA1 treatment, related to **S5B**. n (number of infected cells) = 346 and 312, data points correspond to individual technical replicates from 3 independent experiments. **F.** GAL8 staining in THP-1 macrophages stably expressing GFP-ALG-2 infected or not with Mtb WT or Mtb ΔRD1 for 2.5 h (MOI:2) under the indicated treatments. **G.** Quantification shows the percentage of infected cells showing Mtb-ALG-2 positive structures under indicated treatment. n (number of infected cells) = 146-237, data points correspond to individual technical replicates from 3 independent experiments. **H.** Quantification shows the percentage of Mtb-ALG-2 positive structures positive for GAL8 under BAPTA-AM treatment. n (number of infected cells) = 237 and 218, data points correspond to individual technical replicates from 3 independent experiments. **I.** Quantification shows the percentage of Mtb-GAL8 positive structures positive for ALG-2 under BafA1 treatment. n (number of infected cells) = 237 and 225, data points correspond to individual technical replicates from 3 independent experiments. **Scale bars**: 10 µm (main images), 1 µm (inserted area).

Supplementary Figure 6

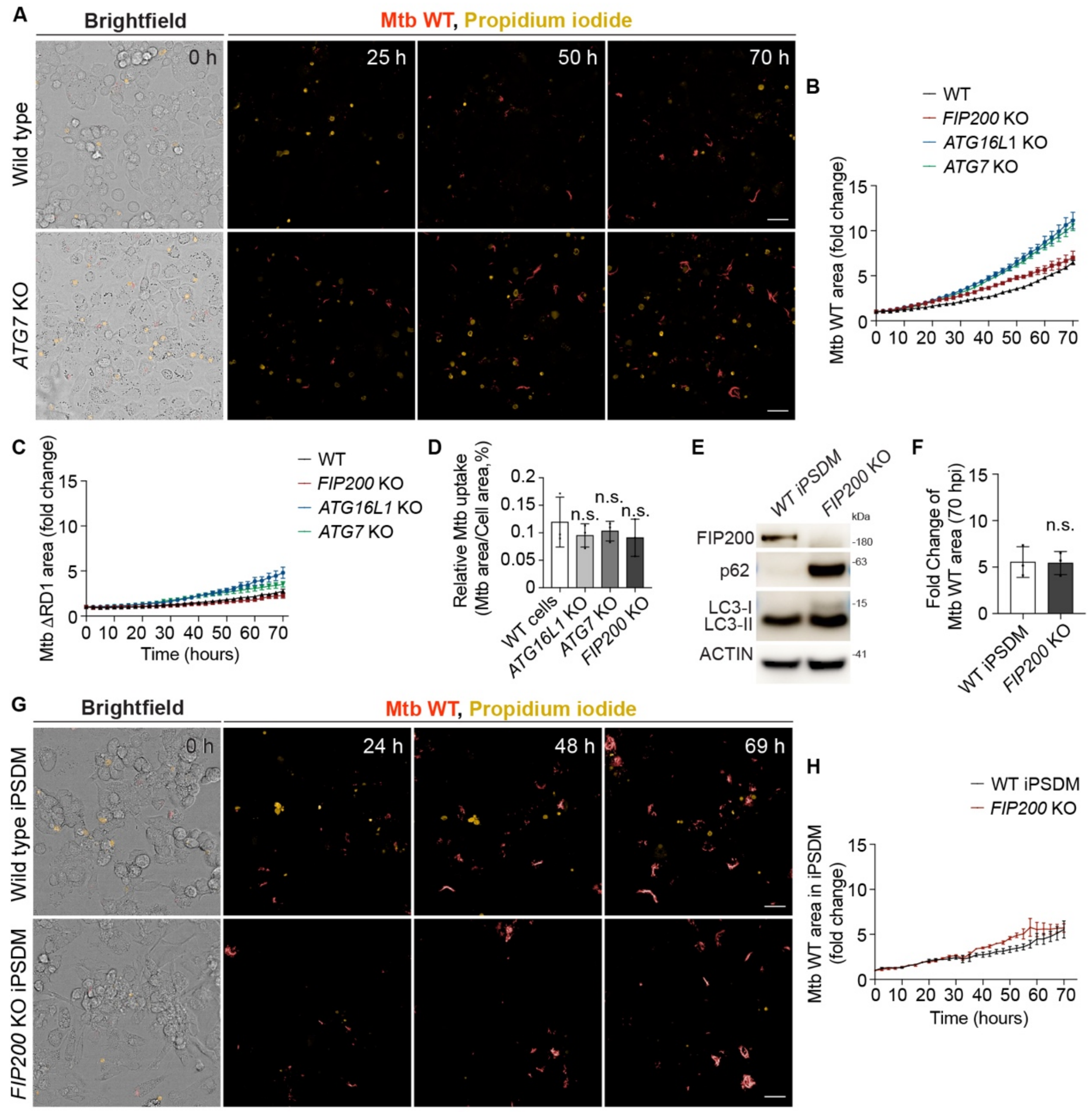

Figure S6: Mtb-LC3-TVS formation restricts Mtb infection

A. Representative micrographs at indicated timepoints of WT and *ATG7* KO THP-1 macrophages infected with Mtb WT (Red) in the presence of PI (pseudo-coloured in yellow). Bright field images shown the localization of macrophages. Data representative from one out of four independent experiments. **B and C.** High-content quantitative analysis of live Mtb WT **(S6B)** replication in indicated THP-1 macrophages related to **Figure 7D and S6A** and Mtb ΔRD1 **(S6C)** replication in indicated THP-1 macrophages. Mtb area was calculated as fold change, relative to Mtb uptake at time 0 h post infection. Data from three independent biological replicates, each of which represents the mean of three technical replicates. **D.** Quantification shows the ratio of Mtb area/cell area at time 0 (related to Figure **7D, 7E**, **S6A and S6B**) in indicated THP-1 macrophages. Data from three

independent biological replicates, each of which represents the mean of three technical replicates. **E.** Western blot analysis of WT and *FIP200* KO iPSDM. **F.** Quantification shows the fold change of Mtb area at 70 h post-infection (hpi) with WT Mtb in WT and *FIP200* KO iPSDM related to **S6G**. Data from three independent biological replicates, each of which represents the mean of three technical replicates. **G.** Representative micrographs at indicated timepoints of WT and *FIP200* KO iPSDM infected with Mtb WT (Red) in the presence of PI (pseudo-coloured in yellow). Bright field images shown the localization of macrophages. Data representative from one out of three independent experiments. **H.** High-content quantitative analysis of live Mtb WT replication in WT and *FIP200* KO iPSDM related to **S6G**. Mtb area was calculated as fold change, relative to Mtb uptake at time 0 h post infection. Data from three independent biological replicates, each of which represents the mean of three technical replicates. **Scale bars**: 50 µm.

Supplementary Figure 7

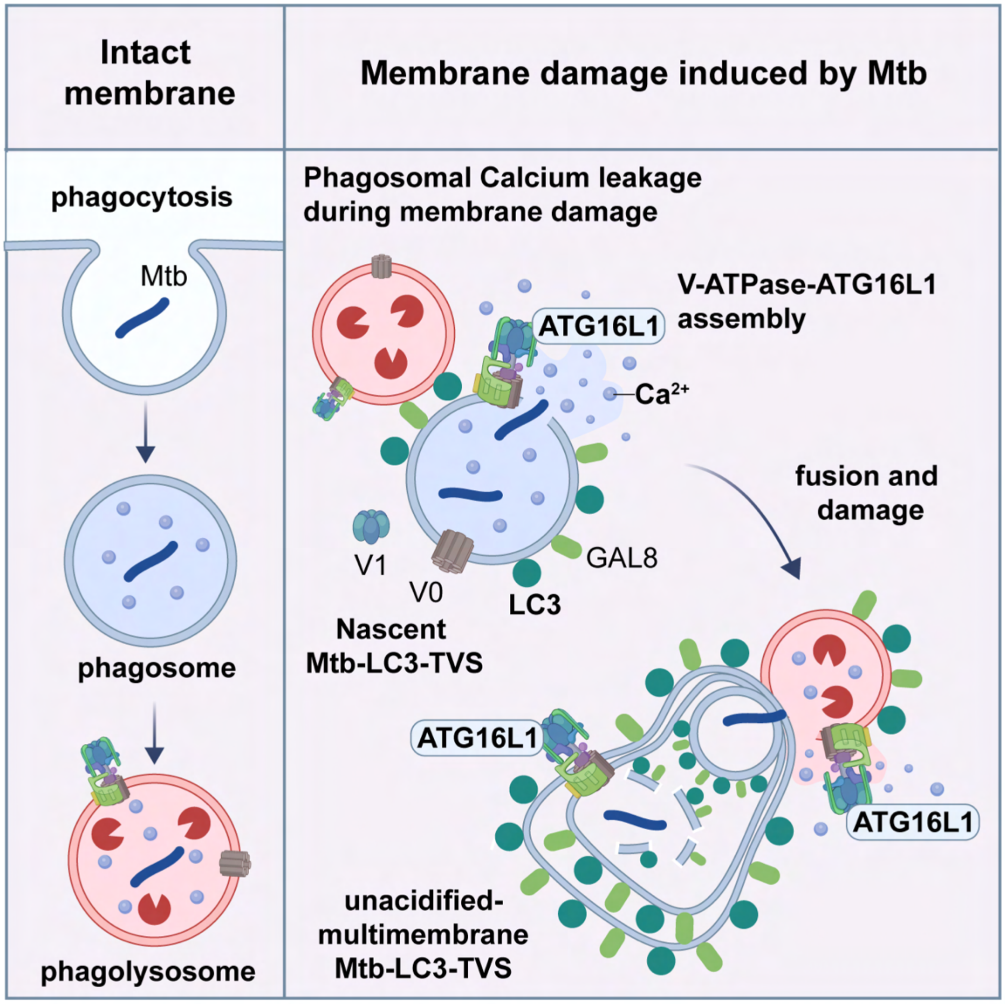

Figure S7: Schematic graph showing Mtb-LC3-TVS formation

After phagocytosis, intact Mtb-containing phagosomes do not trigger LC3-TVS formation and are delivered to acidified lysosomes. However, upon membrane damage, Ca^{2+} leakage from the Mtb phagosome initiates V-ATPase-ATG16L1 assembly and LC3-TVS formation. While nascent Mtb-LC3-TVS structures undergo fusion with lysosomes, Mtb continuously damages the membrane, leading to the formation of unacidified, multilayered Mtb-LC3-TVS structures.

Movie S1: Mtb WT induces phagosome Ca^{2+} leakage
THP-1 macrophages stably expressing LAMP1-GCaMP6f (green) were infected with E2Crimson Mtb WT (magenta) and followed by live cell confocal microscopy with frames every 1- or 2.5-min. Time 0 is the frame for movie start after 2 h Mtb uptake.

Movie S2: Mtb ΔRD1 does not induce phagosome Ca^{2+} leakage
THP-1 macrophages stably expressing LAMP1-GCaMP6f (green) were infected with E2Crimson Mtb ΔRD1 (magenta) and followed by live cell confocal microscopy with frames every 20 sec. Movie is one of the slices of 3 Z slices. Time 0 is the frame for movie start after 2 h Mtb uptake.

Movie S3: Mtb-LC3-TVS do not acidify
iPSDM stably expressing RFP-GFP-LC3B (yellow) were infected with E2Crimson Mtb WT (magenta) and followed by live cell confocal microscopy with frames every 2.5 min. Red only LC3B structures represent acid autolysosomes. Time 0 is the frame for movie start after 2 h Mtb uptake.

Movie S4: LC3-TVS undergo acidification after Mtb segregates
THP-1 macrophages stably expressing GFP-LC3B (green) stained with Lysotracker Red (red) were infected with E2Crimson Mtb WT (magenta) and followed by live cell confocal microscopy with frames every 2 min. Time 0 is the frame for movie start after 2 h Mtb uptake.

Movie S5: Fusion of endolysosomes with Mtb-LC3-TVS provides the multimembrane source
THP-1 macrophages stably expressing GFP-LC3B (green) stained with Lysotracker Red (red) were infected with E2Crimson Mtb WT (magenta) and followed by live cell confocal microscopy with frames every 2 min. Time 0 is the frame for movie start after 2 h Mtb uptake.

Movie S6: Correlative Array tomography reveals complex morphologies at the nanoscale.
Correlative volume EM image showing the alignment of RFP-GFP-LC3B (yellow) and E2Crimson Mtb WT (magenta) fluorescence with a reconstructed array tomography SEM of a *FIP200* KO THP-1 macrophage. Bacteria was segmented (red) to show their relationship to multi-membrane structures in 3D.
